# Uncovering the wide protective responses in *Coffea* spp. leaves to single and superimposed exposure of warming and severe water deficit

**DOI:** 10.3389/fpls.2023.1320552

**Published:** 2024-01-08

**Authors:** Ana P. Rodrigues, Isabel P. Pais, António E. Leitão, Danielly Dubberstein, Fernando C. Lidon, Isabel Marques, José N. Semedo, Miroslava Rakocevic, Paula Scotti-Campos, Eliemar Campostrini, Weverton P. Rodrigues, Maria Cristina Simões-Costa, Fernando H. Reboredo, Fábio L. Partelli, Fábio M. DaMatta, Ana I. Ribeiro-Barros, José C. Ramalho

**Affiliations:** ^1^ Laboratório de Interações Planta-Ambiente e Biodiversidade (PlantStress & Biodiversity), Centro de Estudos Florestais (CEF), Instituto Superior de Agronomia, Universidade de Lisboa, (ISA/ULisboa), Oeiras, Lisboa, Portugal; ^2^ Laboratório Associado TERRA, Instituto Superior de Agronomia, Universidade de Lisboa, (ISA/ULisboa), Lisboa, Portugal; ^3^ Unidade de Investigação em Biotecnologia e Recursos Genéticos, Instituto Nacional de Investigação Agrária e Veterinária, I.P. (INIAV), Oeiras, Portugal; ^4^ Unidade de GeoBiociências, GeoEngenharias e GeoTecnologias (GeoBioTec), Faculdade de Ciências e Tecnologia (FCT), Universidade NOVA de Lisboa (UNL), Caparica, Portugal; ^5^ Centro Univ. Norte do Espírito Santo (CEUNES), Dept. Ciências Agrárias e Biológicas (DCAB), Univ. Federal Espírito Santo (UFES), São Mateus, ES, Brazil; ^6^ Assistência Técnica e Gerencial em Cafeicultura - Serviço Nacional de Aprendizagem Rural (SENAR), Porto Velho, RO, Brazil; ^7^ Setor de Fisiologia Vegetal, Laboratório de Melhoramento Genético Vegetal, Centro de Ciências e Tecnologias Agropecuárias, Universidade Estadual do Norte Fluminense, Rio de Janeiro, Brazil; ^8^ Centro de Ciências Agrárias, Naturais e Letras, Universidade Estadual da Região Tocantina do Maranhão, Maranhão, Brazil; ^9^ Departamento de Biologia Vegetal, Universidade Federal Viçosa (UFV), Viçosa, MG, Brazil

**Keywords:** antioxidant response, climate change, coffee, drought, heat, lipoperoxidation, photoinhibition, sugars

## Abstract

Climate changes boosted the frequency and severity of drought and heat events, with aggravated when these stresses occur simultaneously, turning crucial to unveil the plant response mechanisms to such harsh conditions. Therefore, plant responses/resilience to single and combined exposure to severe water deficit (SWD) and heat were assessed in two cultivars of the main coffee-producing species: *Coffea arabica* cv. Icatu and *C. canephora* cv. Conilon Clone 153 (CL153). Well-watered plants (WW) were exposed to SWD under an adequate temperature of 25/20°C (day/night), and thereafter submitted to a gradual increase up to 42/30°C, and a 14-d recovery period (Rec14). Greater protective response was found to single SWD than to single 37/28°C and/or 42/30°C (except for HSP70) in both cultivars, but CL153-SWD plants showed the larger variations of leaf thermal imaging crop water stress index (CWSI, 85% rise at 37/28°C) and stomatal conductance index (I_G_, 66% decline at 25/20°C). Both cultivars revealed great resilience to SWD and/or 37/28°C, but a tolerance limit was surpassed at 42/30°C. Under stress combination, Icatu usually displayed lower impacts on membrane permeability, and PSII function, likely associated with various responses, usually mostly driven by drought (but often kept or even strengthened under SWD and 42/30°C). These included the photoprotective zeaxanthin and lutein, antioxidant enzymes (superoxide dismutase, Cu,Zn-SOD; ascorbate peroxidase, APX), HSP70, arabinose and mannitol (involving *de novo* sugar synthesis), contributing to constrain lipoperoxidation. Also, only Icatu showed a strong reinforcement of glutathione reductase activity under stress combination. In general, the activities of antioxidative enzymes declined at 42/30°C (except Cu,Zn-SOD in Icatu and CAT in CL153), but HSP70 and raffinose were maintained higher in Icatu, whereas mannitol and arabinose markedly increased in CL153. Overall, a great leaf plasticity was found, especially in Icatu that revealed greater responsiveness of coordinated protection under all experimental conditions, justifying low PI_Chr_ and absence of lipoperoxidation increase at 42/30°C. Despite a clear recovery by Rec14, some aftereffects persisted especially in SWD plants (*e.g*., membranes), relevant in terms of repeated stress exposure and full plant recovery to stresses.

## 1 Introduction

Ongoing climate changes are imposing more frequent and severe environmental limitations on plants due to a warmer and drier environment (Cassia et al., 2018; Lamaoui et al., 2018; IPCC, 2018; Semedo et al., 2018). Abiotic stresses, such as extreme temperatures and drought, cause large economic losses in agriculture, more than halving the yields for major crop species, namely due to their impact on C-assimilation and reproductive structures (Wang et al., 2003; Li et al., 2009; Fábián et al., 2019; Balfagón et al., 2020). These constraints are expected to be aggravated in the coming years, compromising food and cash crop sustainability (Watts et al., 2023), particularly in the tropical regions (IPCC, 2014; IPCC, 2018).

Plants display various stress response mechanisms, encompassing morphological, physiological, biochemical, and molecular levels (Chaves et al., 2009; Osakabe et al., 2014; Lamaoui et al., 2018). Stresses activate cell signaling pathways and responses by promoting specific gene expression, protein and metabolite synthesis to support defense reactions (Jaspers and Kangasjärvi, 2010; Fernandes et al., 2021; Marques et al., 2021; Marques et al., 2022a).

Temperature rise affects all major metabolic processes in C3 plants, stimulating photorespiration and mitochondrial respiration to a larger extent than photosynthesis (Long et al., 2006). Also, chloroplasts are known to be rapidly and deeply affected by environmental constraints, such as heat and drought (Kratsch and Wise, 2000; Mano, 2002). Amid other impacts, heat can inactivate photosystem (PS) II electron acceptor and donor sides, impair electron transport, reduce ribulose-1,5-carboxylase/oxygenase (RuBisCO) activity (Haldimann and Feller, 2004; Balfagón et al., 2020). Additionally, heat induces over-fluidization of membrane lipids, with a potential negative impact on membrane-based events (including thylakoids), modifying hormone and primary and secondary metabolite’s balance, altering protein structure, promoting reactive oxygen species (ROS) overproduction, and inhibiting transcription and translation processes (Wahid et al., 2007; Song et al., 2014).

Stomata closure is one of the first plant responses to water deficit. This limits water loss through transpiration, but simultaneously reduces the leaf heat exchange (consequently increasing its temperature) and restricts CO_2_ diffusion into the leaf, therefore compromising photosynthetic C-assimilation. Additionally, with an exacerbation of water deficit severity, non-stomatal limitations to photosynthesis will additionally take place, with photochemical and biochemical impacts (Chaves et al., 2003; Fahad et al., 2017; Semedo et al., 2021). Furthermore, the decline in the photochemical use of energy often promote oxidative conditions through the overproduction of highly reactive molecules of oxygen and chlorophyll, such as triplet (^3^Chl*) and singlet (^1^Chl) state of Chl, singlet oxygen (^1^O_2_), and superoxide (O_2_^•-^) in PSI and PSII (Logan, 2005; Asada, 2006). The O_2_^•-^ can thereafter result in H_2_O_2_, and afterwards in hydroxyl radical (^•^OH) (Noctor and Foyer, 1998). This in turn further impairs the photosynthetic apparatus, *e.g*., through lipid peroxidation, and inactivation of the Calvin cycle enzymes (Logan, 2005; Halliwell, 2006; Wahid et al., 2007).

Climate change-driven extreme weather events, particularly those that combine rising temperatures and restrained water availability, pose new unique challenges to plants. Such co-occurrence of heat stress and water deficit is an increasingly frequent situation under field conditions, and is much less characterized than the exposure to individual stress conditions (Zandalinas et al., 2021). Therefore, understanding plant performance under stress combinations is crucial to selecting and breeding plant crops, able to maintain yield production under the new predicted climatic conditions (Zandalinas et al., 2018; Balfagón et al., 2020; Zandalinas et al., 2021). The responsive signalling pathways to abiotic stresses often constitute a complex and interconnected network that cross-talks at several levels (Fujita et al., 2006; Yamaguchi-Shinozaki and Shinozaki, 2006). However, each particular stress combination require shared but also specific pathways and processes responses, which play a critical role in the acclimation to multifactorial stress combination (Mittler and Blumwald, 2002; Pandey et al., 2015; Zandalinas et al., 2021). In this sense, impacts/response capability to stress co-occurrence are not directly foreseeable from each single stress condition on morphological and physiological processes, nutrient assimilation and balance, and gene expression (Des Marais et al., 2017; Zandalinas et al., 2018; Zandalinas et al., 2021). In fact, the pre-exposure to drought can even reduce the leaf physiological negative impacts of temperature stress applied afterward (Ramalho et al., 2018; Araújo et al., 2019), and moderate drought can enhance plant tolerance to subsequent heat stress (Zhang et al., 2019). Yet, C-assimilation is greatly hit, as drought and/or heat can affect all photosynthetic components, concurring to drastically depress crop growth and yields (Lamaoui et al., 2018), ultimately affecting global agriculture systems and plant survival (IPCC, 2014; Pandey et al., 2015; Balfagón et al., 2020).

Chloroplasts are a major cellular ROS source due to several oxidative and electron transport reactions, with PSI and PSII reaction centers constituting major generation sites (Logan, 2005). Moreover, ROS formation can be greatly increased when plants are subjected to environmental constraints, particularly when photon energy capture exceeds that required for C-assimilation (Logan, 2005), exacerbating the excitation energy transfer to Chl and O_2_. Additionally, stomata closure will increase the oxygenation function of ribulose-1,5-bisphosphate carboxylase/oxygenase (RuBisCO), boosting H_2_O_2_ production through photorespiration (Bendou et al., 2022). Overall, this leads to an overproduction of highly reactive molecules that can promote lipoperoxidation, PSs and enzymes inactivation, protein oxidation (*e*.*g*., D1), bleaching of pigments (*e*.*g*., P680), and DNA degradation (Feierabend, 2005; Logan, 2005). In accordance, plant tolerance to environmental stresses (*e.g*., drought and/or heat) has been frequently associated with the upregulation of control mechanisms of such highly reactive molecules of Chl and O_2_ (Reddy et al., 2004; Asada, 2006; Zandalinas et al., 2017; Borjas-Ventura et al., 2019; Balfagón et al., 2020; Dumanović et al., 2021). These mechanisms act either through the dissipation of energy excess (*e*.*g*., pigments, pseudocyclic electron transport, photorespiration) that prevent their generation, or by the overexpression of antioxidant enzymes and non-enzymatic antioxidants that scavenge them. Chloroplast antioxidative enzymes such as Cu,Zn-superoxide dismutase (Cu,Zn-SOD), ascorbate peroxidase (APX), and glutathione reductase (GR), are key components of the antioxidative system, often complemented with the extra-chloroplast detoxification systems (*e.g*., catalase, CAT), when H_2_O_2_ diffuses out of chloroplast. SOD-catalysed O_2_^•-^ dismutation produces H_2_O_2_, which is then removed by APX and CAT, avoiding its transformation to ^•^OH through the metal-catalysed Haber-Weiss reaction (Mittler, 2002; Logan, 2005; Smirnoff, 2005; Halliwell, 2006; Dumanović et al., 2021). Non-enzyme compounds contribute also to ROS control (*e.g*., ascorbate (ASC) and α-tocopherol) by directly removing ^1^O_2_, O_2_^•-^, ^•^OH, and lipid peroxyl radicals (Noctor and Foyer, 1998; Karpinski et al., 2002; Smirnoff, 2005; Dumanović et al., 2021). Additionally, the liposoluble carotenoids reduce the amount of energy absorbed by Chl complexes, prevent an over-reduction of the electron transport chain and an over-acidification of the thylakoid lumen, which increases the PSII sensitivity to photodamage. Among them, zeaxanthin removes ^1^O_2_ and performs energy thermal dissipation (reducing ^3^Chl* and ^1^Chl* formation), and carotenes can scavenge highly reactive molecules of Chl and O_2_, protecting light-harvesting complexes (LHCs) and membrane lipids against photooxidation under stress conditions (Mano, 2002; Smirnoff, 2005; Dall'Osto et al., 2012; Demmig-Adams et al., 2022).

Complementary to maintaining an adequate C-partitioning that ensures the supply of energy C-skeletons to support cell metabolism, growth and development, sugars act as key players in stress perception, are part of a short- and a long-distance signalling network, interact with hormones and regulate gene expression and proteomic patterns, namely those related to photosynthetic metabolism, thus playing a key role in crosstalk in abiotic stress pathways (Chaves et al., 2003; Reddy et al., 2004; Rejšková et al., 2007; Saddhe et al., 2021). Sugars (*e.g*., sucrose, glucose, fructose and raffinose family oligosaccharides, RFOs), and sugar alcohols (*e.g*., mannitol) are compatible solutes that contribute to osmotic adjustment and osmotic homeostasis at low leaf water potentials, preserving cell turgor and protein conformation and its biological functions (Quick et al., 1989; Chaves et al., 2003; Rejšková et al., 2007; Lahuta and Górecki, 2011; Ramalho et al., 2014b; Saddhe et al., 2021). Sugars are also associated with membrane stabilization and ROS scavenging (Rejšková et al., 2007; Keunen et al., 2013; Saddhe et al., 2021).

From the 130 species in the *Coffea* genus (Davis and Rakotonasolo, 2021), coffee production and trading are mainly supported by *C. arabica* L. (Arabica coffee), and *C. canephora* Pierre ex A. Froehner (Robusta coffee) (ICO, 2021). Coffee is one of the most traded agricultural commodities, with an income *ca*. USD 200,000 million (ICO, 2021). The entire value chain is estimated to involve 100-125 million people worldwide, based on more than 12.5 million coffee farms. Of these, *ca*. 60% belong to smallholders (Koutouleas et al., 2022), with this crop contributing to support the livelihoods of *ca*. 25 million smallholder farmers in more than 80 tropical producing countries (Bunn et al., 2015; DaMatta et al., 2019; Pham et al., 2019). While successful cultivation of *C. arabica* requires annual mean temperatures around 18-22°C, *C. canephora* requires higher average temperatures (22 up to 30°C), thereby being considered more heat- and less cold-tolerant than *C. arabica* (DaMatta and Ramalho, 2006; Ramalho et al., 2014a). Recent findings demonstrated that elevated air [CO_2_] (eCO_2_) can improve coffee C-assimilation and plant vigor (Ramalho et al., 2013b; DaMatta et al., 2019), C-investment in reproductive structures (Rakocevic et al., 2020), in addition to altering plant growth (Rakocevic et al., 2021), overall associated with the up-regulation of several photosynthetic-related genes (Marques et al., 2020). In fact, model studies suggested that (eCO_2_) can and even increase yields under adequate water supply (Verhage et al., 2017; Rahn et al., 2018), in line with recent filed results (DaMatta et al., 2019). Additionally, eCO_2_ has been reported to greatly mitigate heat (Rodrigues et al., 2016; Scotti-Campos et al., 2019), and drought (Avila et al., 2020; Semedo et al., 2021) impacts on C-assimilation and plant growth. Still, the predicted future global warmer and dryer conditions along this century pose a fundamental challenge (and threat) to coffee producers (Koutouleas et al., 2022). In fact, this crop will increasingly face climate hazards that will provoke strong yield reductions, loss of adequate cultivation areas, altered pest and disease incidence (Magrach and Ghazoul, 2015; Pham et al., 2019; Cassamo et al., 2023; Richardson et al., 2023), and a considerable extinction risk to wild coffee species (Davis et al., 2019; Moat et al., 2019). Although some elite cultivars present greater resilience to environmental stresses than traditionally reported (Dubberstein et al., 2020; Semedo et al., 2021), the impacts might be aggravated by the fact that coffee plantations can last for more than 30 years; thus, actual cropped plants will endure future hasher and uncertain climate conditions (Bunn et al., 2015).

Given the facts described above, coffee’s current level of investment in agricultural R&D should be greatly increased, promoting innovation and knowledge to support the productivity and sustainability of the coffee sector (Maredia and Martínez, 2023). In this context, the uncovering of the underlying mechanisms by which coffee species can deal with the combined exposure to severe drought and heat conditions is of utmost importance for the future of the coffee crop sustainability. *Coffea* spp. plants have been reported to trigger a set of crucial acclimation mechanisms, including photoprotective and antioxidative mechanisms, heat shock proteins (HSP70), cyclic electron flow (CEF) around the PSs. Interestingly, antioxidative mechanisms were found to be commonly triggered across single stress events, being crucial for their resilience to single exposure to cold (Ramalho et al., 2003; Fortunato et al., 2010; Batista-Santos et al., 2011; Ramalho et al., 2013a), heat (Martins et al., 2016; Rodrigues et al., 2016), high irradiance (Ramalho et al., 1998; Ramalho et al., 2000), drought (Lima et al., 2002; Pinheiro et al., 2004; Ramalho et al., 2018; Marques et al., 2022a) and mineral deficiency (Carelli et al., 2006; Pompelli et al., 2010; Ramalho et al., 2013a). Here, we present a comprehensive analysis of the response mechanisms to both single and combined drought and heat conditions, in two cropped cultivars of the two major coffee producing species. This analysis encompassed antioxidative (*e.g*., enzymes) and other complementary molecules (*e.g*., HSP70, photoprotective carotenoids, sugars), and their relationship with physiological impacts (*e.g*., PSII photoinhibition status, thermal imaging, membrane selectivity) and plant resilience. Our novel results allow to advance knowledge previously obtained (Dubberstein et al., 2020) on the mechanistic basis of coffee acclimation capability to future harsh environments in which these stresses superimposition will be increasingly frequent.

## 2 Materials and methods

### 2.1 Plant material and exposure to environmental conditions

#### 2.1.1 Plant material and growth conditions

Two widely used cultivars, *Coffea canephora* Pierre ex A. Froehner cv. Conilon Clone 153 (CL153) and *C. arabica* L. cv. Icatu Vermelho (Icatu, an introgressed variety resulting from a cross of *C. canephora* and *C. arabica* cv. Bourbon Vermelho, then further crossed with *C. arabica* cv. Mundo Novo), were evaluated following the experimental design described earlier (Dubberstein et al., 2020). A total of 32 plants were grown since the seedling stage, during 7 years in 80 L pots, in two walk-in growth chambers (EHHF 10000, ARALAB, Portugal) under controlled temperature (25/20°C, day/night, ± 1°C), irradiance (*ca*. 700-800 μmol m^-2^ s^-1^ at the upper canopy), relative humidity (70 ± 2%), photoperiod (12 h), and air [CO_2_] (380 ± 5 μL CO_2_ L^-1^). Each potted plant was grown in a substrate consisting of a mixture of soil, peat, and sand (3:1:3, v/v/v), with a pH 6.5, and were maintained under adequate soil moisture by watering the plants every two days (until applying the water treatments), and without restrictions in nutrients (see Ramalho et al., 2013a; Ramalho et al., 2013b), or root development (as judged by visual examination at the end of the experiment after removing the plants from their pots). Water deficit and heat conditions (see below) were gradually (to allow plant acclimation) and sequentially imposed in 8 plants (with close size and shape, especially within each genotype) per each treatment and cultivar.

#### 2.1.2 Severe drought imposition

Water treatments were established under adequate temperature (25/20°C, day/night), considering well-watered (WW) or single severe water deficit (SWD) conditions, which represented approximately 80 or 10% of maximal pot water availability, respectively (Ramalho et al., 2018). Overall, SWD was reached after two weeks by partially withholding irrigation (through a partial reposition every two days of water that was lost in each pot) when leaf predawn water potential (*Ψ_pd_*) was below -3.7 MPa. WW plants were maintained fully irrigated, with *Ψ_pd_* close to or above -0.35 MPa. After reaching those *Ψ_pd_* values in SWD plants, these conditions were maintained for another five days before the onset of temperature rise. These water conditions were maintained afterwards along the gradual temperature rise. Water status evaluation was performed by daily visual observation of the leaf/plants (wilting status), and confirmed by frequent measurements of leaf water potential at predawn (when it equilibrates with soil water potential), as stated in 2.3 (see below).

#### 2.1.3 High temperature imposition

After establishing the water availability conditions, WW and SWD plants were submitted to a temperature increment of 0.5°C day^-1^ (of the diurnal temperature) from 25/20°C up to 42/30°C, with an additional 5 days of stabilization at the temperatures of 31/25°C, 37/28°C and 42/30°C to enable the programmed evaluations and sampling. Finally, control conditions were reestablished (25/20°C and full irrigation), with the potential recovery being monitored over 14 days (Rec14).

Overall, the SWD plants reached the desired *Ψ_pd_* within 14 days upon gradual drought imposition and were kept in these conditions another five days, before the onset of temperature rise up to 42/30°C (49 days). Afterwards, plants were watered and the control temperature re-established, followed by a recovery period (14 d). The entire long-term experiment lasted 82 days.

### 2.2 Physiological and biochemical evaluations

Determinations were carried out on newly matured leaves from the upper (well-illuminated) third part of the plants usually at 25/20, 31/25, 37/28, 42/30°C and Rec14. Unless otherwise stated, evaluations or samplings were performed after *ca*. 2 hours of illumination, under photosynthetic steady-state conditions.

Evaluations were performed *in vivo* (*Ψ_pd_*, thermal imaging, PSII photoinhibition status, electrolyte leakage, antioxidant enzymes), or leaf material was collected, immediately flash frozen in liquid N_2_ and stored at -80°C, being finely powdered in liquid N_2_ prior to analysis (MDA, non-enzyme protective molecules, sugars). Leaf tissue extractions were performed by using ice-cold mortar and pestles, as well as cold homogenizing solutions.

### 2.3 Water status monitoring

Leaf water potential was determined at predawn (*Ψ_pd_*) immediately after leaf excision (Schölander et al., 1965) using a pressure chamber (Model 1000, PMS Instrument Co., Albany, OR, USA). Monitoring was done, usually every three days, although only the data at the main points for all data collection (along temperature rise and recovery periods) are presented.

### 2.4 Thermal imaging analysis

Thermal images were acquired with a thermal imager (GF300, FLIR Systems, Wilsonville, OR, USA) and processed using a Thermal Cam Explorer software (FLIR Systems), following the procedures and formulae previously given (Grant et al., 2007; Jones and Grant, 2016), as described in detail for coffee plants (Semedo et al., 2021). The thermal camera operates in the wavebands 7.5-13 µm, has a thermal resolution of 0.06°C, an accuracy of ± 2°C, and produces images of 640 x 480 pixels, with a field of view of 45°. Images were taken 1.5 m from the canopies, resulting in a spatial resolution of approximately 1 mm. Briefly, images were corrected for spatial calibration drift by subtracting corresponding reference images of an isothermal surface. The canopy was imaged using reference leaves to simulate fully closed and fully open stomata. Reference leaves representing fully closed stomata had both sides covered with petroleum jelly (Vaseline) to obtain the dry temperature (T_dry_). Their leaf counterparts, representing fully open stomata, were sprayed with water using a hand spray bottle to maintain maximal moisture level and to obtain the wet temperature (T_wet_). The temperatures of the reference leaves (T_wet_ and T_dry_), as well as the actual leaf temperature (T_leaf_), were used to calculate the stomatal conductance index [I_G_ = (T_dry_ - T_leaf_)/(T_leaf_ - T_wet_)] that is theoretically proportional to stomatal conductance to water vapour (g_s_). Additionally, following the Idso concept (Idso et al., 1981), it was also calculated the crop water stress index [CWSI = (T_dry_ - T_leaf_)/(T_dry_ - T_wet_)] (Grant et al., 2007), which varies from near 0 (representing a fully transpiring leaf/crop with stress absence) to 1 (representing a non-transpiring leaf/crop under severe stress).

### 2.5 Photosystem II photoinhibition

Chlorophyll (Chl) *a* fluorescence measurements were performed using a PAM-2000 system (H. Walz, Effeltrich, Germany). Data were taken in the same leaf samples and the same conditions previously described (Dubberstein et al., 2020). This allowed to obtain the maximal photochemical efficiency of PS II (F_v_/F_m_), evaluated in overnight dark-adapted leaves, and the actual PSII photochemical efficiency (F_v_’/F_m_’), evaluated under photosynthetic steady-state conditions (please see Table 2 of Dubberstein et al. (2020) for access to F_v_/F_m_ and F_v_’/F_m_’ values). The photosystem II (PSII) functional status was assessed through the photoinhibition indices already used for coffee leaves (Martins et al., 2016) considering: (A) dynamic photoinhibition (*PI_Dyn_*), representing the decline in F_v_/F_m_, which is fully reversible at night, being measured as the percentage reduction of F_v_’/F_m_’ compared to F_v_/F_m_ at each treatment, in relation to the maximum F_v_/F_m_ of the whole experiment; (B) chronic photoinhibition (PI_Chr_), reflecting the percentage reduction in F_v_/F_m_ of each treatment in relation to the maximum F_v_/F_m_ recorded in the entire experiment; (C) total photoinhibition (*PI_Tota_*_l_ = *PI_Chr_* + *PI_Dyn_*).

### 2.6 Membrane impact assessment

#### 2.6.1 Membrane leakage

Cellular membrane selectivity/integrity was evaluated as described for coffee leaves (Scotti-Campos et al., 2019). Briefly, 10 freshly cut leaf discs (0.5 cm^2^ each) were immediately rinsed three times and subsequently left to float on 15 mL deionized water (24 h, 20°C), after which the sample conductivity resulting from electrolyte leakage was measured (Crison GLP31, Crison Instruments, S.A., Barcelona, Spain). Afterwards, samples were exposed to 90°C for 2 h, followed by cooling to 20°C, when total conductivity was assessed. Membrane leakage was expressed as the percentage of total conductivity.

#### 2.6.2 Membrane lipoperoxidation

Lipid oxidation evaluation was based on the malondialdehyde (MDA) or thiobarbituric acid reactive-substances (TBARS) assay (Hodges et al., 1999; Landi, 2017), using *ca*. 150 mg (FW) of powdered frozen leaves. MDA equivalent contents were quantified at Abs_532 nm_, using the equations proposed by Hodges et al. (1999), after subtracting the non-specific value at Abs_600 nm_ for nonspecific turbidity, the Abs_532 nm_ associated with interfering compounds that absorb at this wavelength, and the Abs_440 nm_ for the correction of potential interference generated by sucrose, and using the molar extinction coefficient of 157 mmol L^-1^ cm^-1^ for MDA calculations.

### 2.7 Maximum apparent activities of antioxidant enzymes

#### 2.7.1 Chloroplast superoxide dismutase

For superoxide dismutase (Cu,Zn-SOD, EC 1.15.1.1) activity, 4 g FW of leaf tissue was homogenized in 25 mL of 20 mM Tricine-KOH (pH 8.0) extraction buffer, containing 0.4 M sucrose, 10 mM NaCl and 30 mM ascorbate. The samples were then filtered through 8 layers of cheesecloth and centrifuged (3000 g, 5 min, 4°C). The pellet was resuspended in 5 mL of 100 mM TRIS-HCl (pH 8.0), containing 0.1 mM EDTA, and 0.3% (v/v) Triton X-100. After a new centrifugation (15000 g, 15 min, 4°C) the supernatant was used for the activity assays at 25°C based on the inhibition rate of ferricytochrome *c*, at 550 nm, following the method of McCord and Fridovish (1969), and previously described in detail for coffee leaves (Ramalho et al., 1998). One unit of Cu,Zn-SOD was defined as the amount of enzyme needed to cause a 50% inhibition in the Cyt *c* reduction rate under the assay conditions.

#### 2.7.2 Chloroplast ascorbate peroxidase

Ascorbate peroxidase (APX, EC 1.11.1.11) activity was assessed following Nakano and Asada (1981). Chloroplast isolation was done by homogenizing *ca*. 3 g FW leaf tissues in 25 mL of 50 mM HEPES (pH 7.6) extraction buffer, containing 0.35 M sorbitol, 1 mM EDTA, 0.4% (w/v) BSA, and 2 mM mercaptoethanol. The samples were then filtered through 8 layers of cheesecloth and centrifuged (3000 g, 5 min, 4°C). The pellet was resuspended in 5 mL of 25 mM HEPES (pH 7.6). APX activity was assessed based on ascorbate consumption (at 290 nm, 120 s, 25°C), and assuming an extinction coefficient of 2.8 mM^-1^ cm ^-1^ for calculations, as referred previously for coffee leaves (Ramalho et al., 1998).

#### 2.7.3 Chloroplast glutathione reductase

For glutathione reductase (GR, EC 1.6.4.2) activity, 4 g FW of leaf tissues were homogenized in 25 mL of 100 mM TRIS-HCl (pH 6.9) extraction buffer, containing 0.4 M sucrose, 10 mM ascorbate, and 2% (w/v) soluble PVPP (Foster and Hess, 1980). The samples were filtered through 8 layers of cheesecloth and centrifuged (3000 g, 5 min, 4°C). The chloroplast pellet was ressuspended in 4 mL of 150 mM HEPES (pH 8.0), containing 1 mM EDTA, and 0.2% (v/v) Triton X-100, and centrifuged again (8000 g, 10 min, 4°C) (Foyer et al., 1995). The supernatant was used for the activity assays, at 25°C according to Schaedle and Bassham (1977), in 1.775 ml reaction buffer solution of 50 mM TRIS-HCl, pH 7.5, containing 3 mM MgCl_2_, and 200 µL, 0.5 mM GSSG, and 0.15 mM NADPH. After adding 25 ml of the supernatant, Abs_340nm_ decrease, corresponding to the NADPH oxidation rate, was followed for 2 min. To avoid overestimation of GR activity, a correction for NADPH oxidation independent of GSSG was performed by adding 25 ml extract to the reference cuvette and omitting GSSG. Solutions of NADPH between 20 and 400 mL^-1^ were used to obtain a standard curve.

#### 2.7.4 Cellular catalase

The assay for catalase (CAT, EC1.11.1.6) activity was based on Havir and McHale (1987), using *ca*. 200 mg FW of leaf material, homogenized in 5 mL of 100 mM Na-Phosphate (pH 7.0) extraction buffer, containing 0.5 mM mercaptoethanol, and adding 2% (w/v) soluble PVPP to each homogenate. The samples were filtered through eight layers of cheesecloth, after which the homogenate was used for enzyme assays. Enzyme activity was estimated by following the rate of H_2_O_2_ consumption for 200 s, at 240 nm, by adding a 200 µL aliquot of the enzyme extract, to the reaction mixture of 25 mM Na–Phosphate buffer (pH7.0), and 50 µL of 50 mM H_2_O_2_.A freshly made H_2_O_2_ standard curve was used for quantification (Fortunato et al., 2010).

### 2.8 Non-Enzymatic protective molecules

#### 2.8.1 Ascorbate determination

Ascorbate quantification was performed based on Romero-Rodrigues et al. (1992), after minor changes described for coffee leaves coffee leaves (Ramalho et al., 2018), using *ca*. 100 mg FW of leaf material. A reverse phase HPLC analysis using a C18, Spherisorb ODS 2 column (250 mm × 4.6 mm; 5 μm pore size), end-capped, and detection at 254 nm, by a UV-Vis detector (model 440, Waters Millipore Associates, USA) was applied. The elution of 20 µL aliquots was performed with H_2_O at pH 2.2 (by adding H_2_SO_4_) for 15 min at a flow rate of 0.4 mL min^-1^. For quantification, a standard curve of ascorbate was used.

#### 2.8.2 Quantification of heat shock protein 70 kDa

The heat shock protein 70 kDa (HSP70) content was assessed based on Njemini et al. (2003), as described in detail for leaf coffee samples (Martins et al., 2016), using samples of 100 mg FW of powdered frozen leaves. After sample processing HSP70 assays were performed through an Enzyme-Linked Immunosorbent Assay (ELISA) using Flat-bottomed micro-ELISA plates (Costar, Corning, NY, USA), with readings at Abs_405nm_ by a microplate absorbance reader (iMark, Bio-Rad, Japan). HSP70 quantification was performed using the absorbance of purified HSP70 protein. The total soluble protein contents were assessed following Bradford (1976) using bovine serum albumin (BSA) as a standard.

#### 2.8.3 Photosynthetic pigment evaluations

Carotenoid (Car) contents were assessed using frozen leaf discs (each 0.5 cm^2^) cut under the growth chamber conditions. All procedures were carried out exactly as previously described in detail for leaf coffee samples (Vinci et al., 2022), with minor adjustments. Briefly, after extraction in aqueous 90% acetone (v/v), and sample processing and filtration (13 mm, nylon), the separation of the pigments was achieved through a reverse phase HPLC analysis using an end-capped C18, 5 µm Spherisorb ODS 2 column (250 mm × 4.6 mm). Detection was performed at 440 nm using an HPLC system (Beckman, System Gold, Tulsa, OK, USA) coupled to a diode detector (Model 168, Beckman). Identification and quantification of each compound was achieved using individual pigment standards. The de-epoxidation state (DEPS), involving the components of the xanthophyll cycle, was calculated as DEPS = (zeaxanthin (Z)+0.5 antheraxanthin (A))/(violaxanthin (V)+A+Z).

Chlorophyll (Chl) content was obtained from spectrophotometric measurements of the same homogenates, diluted to 80% acetone and using the formulae of Lichtenthaler (1987).

#### 2.8.4 Non-structural carbohydrate quantification

Soluble sugars were determined in approximately 150 mg of powdered frozen material, based on the method of Damesin and Lelarge (2003), as described in detail for coffee leaf material (Ramalho et al., 2013b). Briefly, after processing the samples, a 50 µL aliquot was injected into an HPLC system equipped with a refractive index detector (Model 2414, Waters, East Lyme, CT, USA), and the separation of sugars was performed using a Sugar-Pak 1 column (300 x 6.5 mm, Waters) at 90°C, with H_2_O (containing 50 mg EDTA-Ca L^-1^ H_2_O) as the eluent, at a flow rate of 0.5 mL min^-1^. To resolve potential non-pure peaks, another 20 µL aliquot of each sample was injected through a DionexCarboPac PA1 analytical column (4 x250 mm, Thermo Scientific, Waltham, MA, USA) coupled to a DionexCarboPac PA1 Guard (4 × 50 mm) at 20°C. Ultrapure water and 300 mM NaOH were used as eluents (water from 0 to 50 min; NaOH from 50 to 65 min; and water from 65 to 80 min for re-equilibration), at a 1 mL min^-1^ flow rate. Standard curves were used for the quantification of each sugar.

Starch quantification was performed according to Stitt et al. (1978) with some changes exactly as described for coffee leaf material (Ramalho et al., 2013b), after the breakdown of starch to glucose, which was then enzymatically determined, with spectrophotometric readings at Abs_340nm_.

### 2.9 Experimental design and statistical analysis

Samples from CL153 and Icatu cultivars were independently subjected to eight treatment combinations, forming a 2 x 4 factorial consisting of two water availability levels (WW or SWD) and several temperatures (usually 25/20, 37/28, 42/30°C and Rec14) under a completely randomized design within the growth chambers, with eight plants per treatment, with sampling ranging from four to six biological replicates, depending on the trait.

Physiological and biochemical data were analysed using two-way ANOVA to evaluate the differences between the two water availability levels, between the temperature treatments, and their interaction, followed by a posteriori Tukey’s HSD test for mean comparisons. Data analysis was performed using STATISTICA v7.0 (StatSoft). A 95% confidence level was adopted for all tests, which were performed always independently for each cultivar. Data analysis was performed using STATISTICA v7.0 (StatSoft, Hamburg, Germany).

## 3 Results

**Figure 1:**
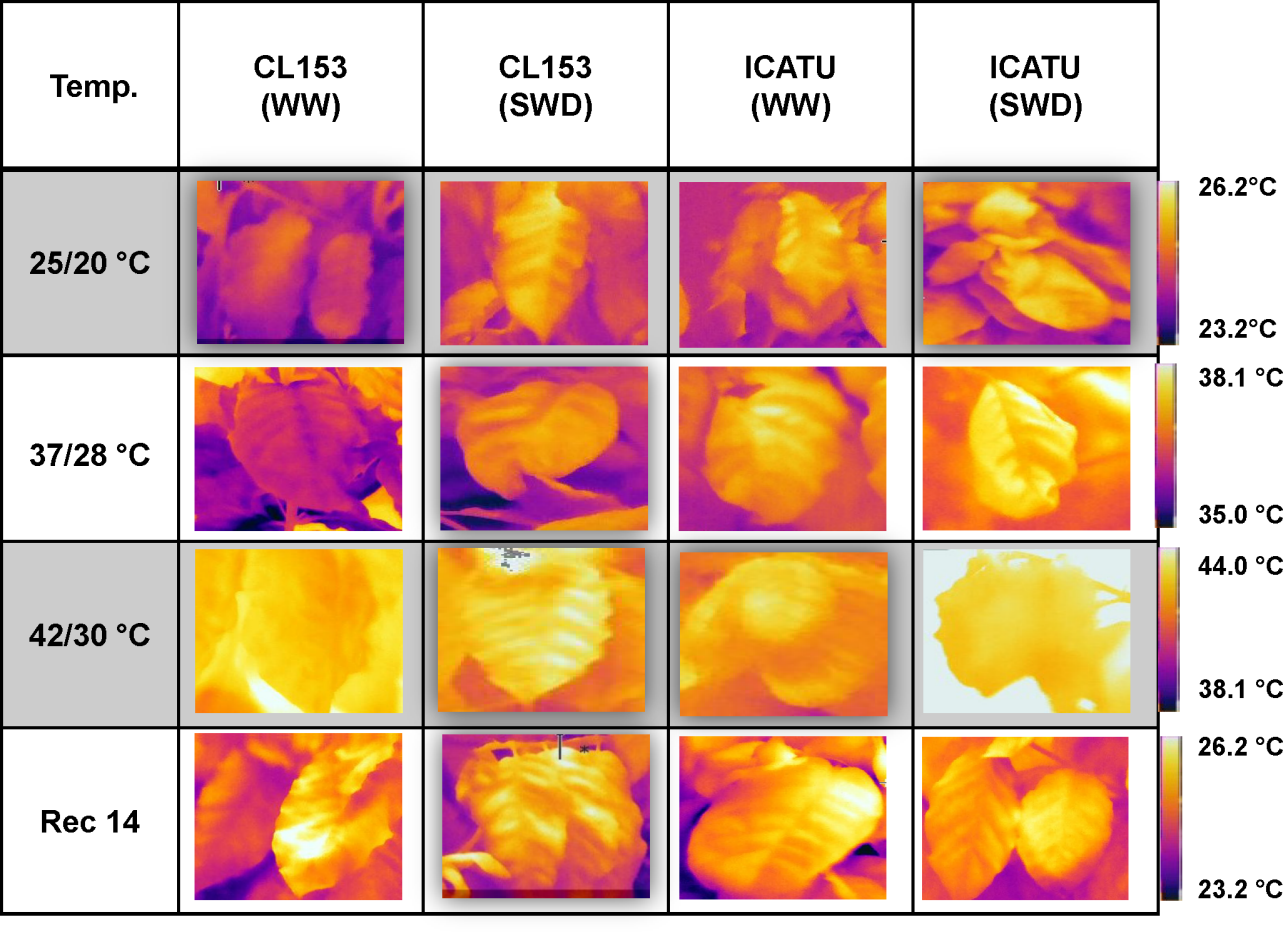
Example of thermal images from a representative coffee leaf from the upper third (illuminated) part of each plant of *Coffea canephora* cv. Conilon Clone 153 (CL153) and *C. arabica* cv. Icatu plants under well-watered conditions (WW) or submitted to severe water deficit (SWD), followed by a gradual temperature increase from (25/20°C, day/night), to 42/30°C, and a recovery period of 14 days (Rec14). The temperature scale at the right of the image range from cooler (purple) to hotter (orange/yellow) but changes accordingly with each temperature condition.

Water stress reached severe levels as shown by the *Ψ_pd_* below -3.7 MPa in SWD plants at 25/20°C, in both cultivars. In turn, *Ψ_pd_* values were not significantly altered by the two highest temperatures under the imposition of this single stress, showing that temperature *per se* did not alter the leaf water status in WW plants (see also Dubberstein et al., 2020). However, the combined stress exposure (SWD at 37/30°C and 42/30°C) tended to minimal *Ψ_pd_* values between -4.1 and -4.5 MPa, in both cultivars. Such SWD plants showed an almost full recovery as compared to their respective controls, just 4 days after simultaneous rewatering and return to 25/20°C (data shown in Dubberstein et al., 2020; **Figure 1**).

### 3.1 Thermal imaging evaluation

**Figure 2:**
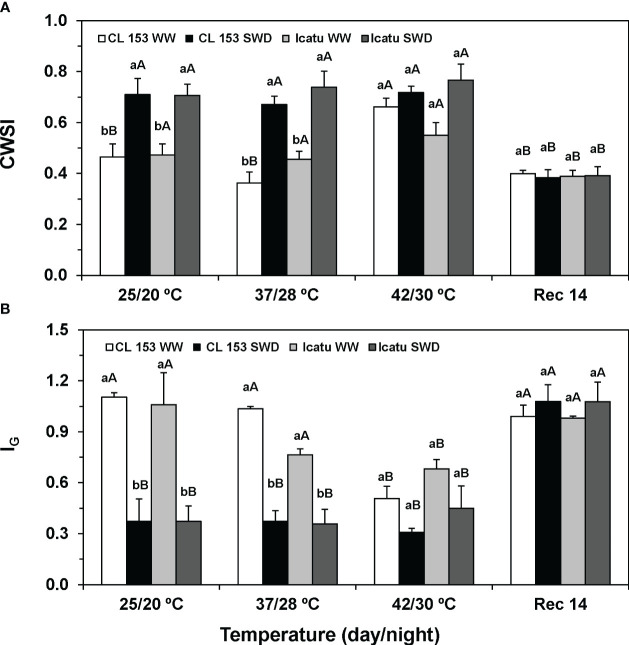
Changes of the **(A)** crop water stress index (CWSI), and **(B)** stomatal conductance index (I_G_), calculated from leaves of *Coffea canephora* cv. Conilon Clone 153 (CL153) and *C arabica* cv. Icatu plants under well-watered conditions (WW) or submitted to severe water deficit (SWD), followed by a gradual temperature increase from (25/20°C, day/night), to 42/30°C, and a recovery period of 14 days (Rec14). For each parameter, different letters after the mean values ± SE (n=5) express significant differences among temperature treatments for the same water level (A–D), or among water availability levels for each temperature treatment (a, b), always separately for each cultivar.

Thermal imaging photos illustrate an increased leaf temperature under single drought (25°C) or heat (WW) (**Figure 1**). Stress superimposition pointed to an even greater leaf temperature at 37/28°C, but at the harshest temperature (42/30°C), no differences were visible regardless of water conditions. Then, after two weeks under control conditions (Rec14), only a partial recovery was observed, similar for both water conditions. These findings globally agreed with CWSI and I_G_ indexes that showed opposite patterns of variation. Single SWD or heat (42/30°C) significantly altered CWSI (rise) and I_G_ (decline) (**Figure 2**). Only Icatu-WW plants maintained unaffected CWSI values at the highest temperature, as compared with their values at 25/20°C. Again, clear differences between WW and SWD plants were observed up to 37/28°C in these indexes, which were markedly attenuated by 42/30°C. Maximal variations of CWSI (85% rise in SWD-37/28°C) and I_G_ (66% decline in SWD-25/20°C) where found in CL153, as compared to their WW counterparts at the same temperature, that is, CSWI almost doubled, whereas I_G_ represents less than half of the control values.

Contrary to thermal images, at Rec 14, the plants recovered to values similar to their controls at the beginning of the experiments (25/20°C, WW). With the mentioned exception in Icatu-WW, both cultivars showed similar pattern of variation of CSWI and I_G_.

### 3.2 PSII photoinhibition status

Single stress conditions impacted the photoinhibition state of photosystem (PS) II, but differently between cultivars (**Table 1**). SWD significantly promoted *PI_Ch_* only in CL153, whereas *PI_Dyn_* and *PI_Tot_* significantly rose in both cultivars. Single heat exposure did not alter all indexes up to 39/30°C (WW plants), but *PI_Chr_* and *PI_Tot_* clearly rose in both cultivars (greater in Icatu) by 42/30°C. Under stress combination (SWD, 42/30°C) only *PI_Chr_* increased, in comparison to their SWD plants at 25/20°C, but in Icatu the *PI_Chr_* value was smaller than that in their WW counterparts at 42/30°C. Notably, *PI_Dyn_* was greater (and reflected in *PI_Total_*) in SWD than in WW plants of both cultivars at 42/30°C, suggesting reinforced protective mechanisms.

**Table 1:** Changes in the leaf chlorophyll *a* fluorescence parameters dynamic photoinhibition (*PI_Dyn_*), chronic photoinhibition (*PI_Chr_*) and total photoinhibition (*PI_Total_*), in *Coffea canephora* cv.

Genotype	Water	Temperature (day/night)									
		25/20 °C	28/23 °C	31/25 °C	34/28 °C	37/28 °C	39/30 °C	42/30 °C	Rec4	Rec10	Rec14
PI_Chr_											
**CL 153**	**WW**	5.7 ± 0.7 bBCD	3.4 ± 0.6 bBCD	3.1 ± 0.3 bCD	3.0 ± 0.3 bD	3.1 ± 0.6 bD	6.4 ± 0.6 bBCD	22.3 ± 2.6 aA	11.9 ± 1.0 bAB	11.1 ± 1.4 bBC	7.8 ± 0.8 bBCD
	**SWD**	15.4 ± 2.5 aBCD	12.4 ± 3.4 aBCD	11.0 ± 2.2 aCD	5.9 ± 0.8 aD	6.8 ± 0.9 aD	9.3 ± 2.2 aBCD	23.3 ± 2.5 aA	18.4 ± 0.6 aAB	16.0 ± 1.5 aBC	10.7 ± 0.6 aBCD
**Icatu**	**WW**	7.8 ± 0.5 aB	8.8 ± 0.6 aB	6.4 ± 0.4 aB	5.8 ± 0.9 aB	9.6 ± 0.8 aB	4.8 ± 0.2 aB	39.4 ± 6.2 aA	11.8 ± 1.2 aB	10.2 ± 0.9 aB	12.5 ± 1.7 aB
	**SWD**	6.9 ± 1.0 aB	3.8 ± 0.5 aB	4.2 ± 0.7 aB	6.9 ± 0.7 aB	9.3 ± 1.7 aAB	7.9 ± 0.7 aAB	18.4 ± 1.0 bA	13.6 ± 1.1 aAB	12.4 ± 0.5 aAB	12.3 ± 0.8 aAB
PI_Dyn_											
**CL 153**	**WW**	21.7 ± 2.4 bA	20.7 ± 4.0 bA	21.3 ± 2.8 bA	26.1 ± 3.0 bA	16.8 ± 1.7 bA	18.7 ± 3.5 bA	27.4 ± 3.4 bA	27.7 ± 5.3 aA	20.6 ± 4.9 aA	19.4 ± 2.6 aA
	**SWD**	41.4 ± 2.9 aA	29.8 ± 2.2 aA	33.7 ± 3.6 aA	38.3 ± 1.9 aA	38.6 ± 3.8 aA	36.6 ± 4.8 aA	34.9 ± 2.9 aA	27.9 ± 1.6 aA	21.3 ± 3.6 aA	24.4 ± 2.9 aA
**Icatu**	**WW**	19.9 ± 2.5 bA	19.8 ± 3.3 bA	11.9 ± 2.3 bA	23.5 ± 3.8 bA	16.2 ± 2.1 bA	25.8 ± 1.8 bA	26.1 ± 4.9 bA	20.8 ± 2.4 aA	20.7 ± 2.3 aA	10.7 ± 2.7 bA
	**SWD**	38.4 ± 2.6 aAB	39.6 ± 3.0 aAB	40.3 ± 4.2 aA	41.6 ± 4.0 aA	37.4 ± 3.3 aAB	44.2 ± 3.1 aA	40.2 ± 2.9 aAB	24.4 ± 2.6 aBC	19.4 ± 2.0 aC	21.4 ± 2.6 aC
Total PI											
**CL 153**	**WW**	27.4 ± 2.8 bAB	24.1 ± 4.4 bB	24.4 ± 2.9 bB	29.2 ± 3.1 bB	19.9 ± 1.9 bB	25.1 ± 3.6 bB	49.7 ± 3.4 bA	39.6 ± 5.3 bAB	31.6 ± 4.7 bB	27.2 ± 2.9 bB
	**SWD**	56.7 ± 4.5 aAB	42.2 ± 4.3 aB	44.7 ± 4.7 aB	44.1 ± 2.3 aB	45.5 ± 4.2 aB	45.9 ± 6.9 aB	58.2 ± 3.1 aA	46.2 ± 1.4 aAB	37.2 ± 3.7 aB	35.1 ± 2.8 aB
**Icatu**	**WW**	27.7 ± 2.6 bB	28.6 ± 3.4 bB	18.2 ± 2.2 bB	29.3 ± 3.8 bB	25.8 ± 1.7 bB	30.6 ± 1.9 bB	65.5 ± 6.3 aA	32.6 ± 1.8 aB	30.9 ± 2.3 aB	23.2 ± 2.2 aB
	**SWD**	45.3 ± 2.4 aABC	43.4 ± 3.1 aABC	44.5 ± 4.8 aABC	48.4 ± 4.3 aBCD	46.7 ± 2.9 aABC	52.1 ± 3.4 aAB	58.6 ± 2.8 aA	38.1 ± 3.2 aBC	31.8 ± 2.3 aC	33.7 ± 2.4 aC

Conilon Clone 153 (CL153) and Coffea arabica cv. Icatu plants, submitted to well-watered (WW) and severe water deficit (SWD), followed by a temperature increase from (25/20°C, day/night), to 42/30°C, and recovery periods of 4 (Rec4), 7-10 (Rec10) and 14 (Rec14) days. For each parameter, different letters after the mean values ± SE (n=6) express significant differences among temperature treatments for the same water level (A, B, C, D), or among water availability levels for each temperature treatment (a, b), always separately for each cultivar.

At Rec4 all photoinhibition indexes strongly recovered in both cultivars as compared to their values at 42/30°C. Still, from Rec4 until Rec14, the CL153-SWD plants maintained higher values of *PI_Chr_* and *PI_Tot_* than their WW counterparts, and both cultivars displayed a tendency to greater values of these indexes as compared with the initial control values (25/20°C, WW), thus suggesting a moderate persistence of some aftereffects.

### 3.3 Stress impacts on membranes

**Figure 3:**
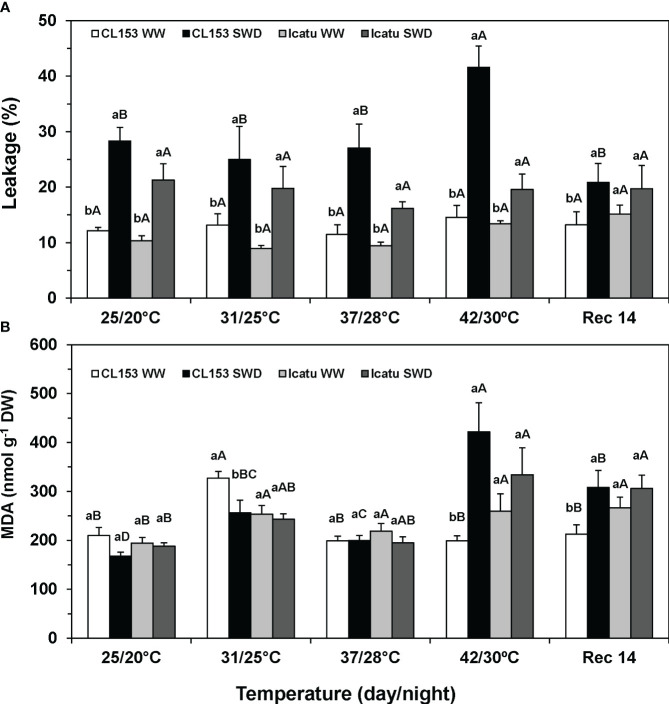
Assessment of leaf membrane integrity **(A)** through electrolyte leakage, and **(B)** lipoperoxidation (associated with MDA content) in *Coffea canephora* cv. Conilon Clone 153 (CL153) and *Coffea arabica* cv. Icatu plants under well-watered conditions (WW) or submitted to severe water deficit (SWD), followed by a gradual temperature increase from (25/20°C, day/night), to 42/30°C, and a recovery period of 14 days (Rec14). For each parameter, different letters after the mean values ± SE (n=5) express significant differences among temperature treatments for the same water level (A–D), or among water availability levels for each temperature treatment (a, b), always separately for each cultivar.

Regarding single stress imposition, only SWD significantly increased membrane permeability, especially in CL153 (**Figure 3A**). In contrast, drought did not affect the lipoperoxidation level (assessed through MDA content) in both cultivars, whereas heat promoted a transient MDA rise in CL153-WW plants at 31/25°C, and moderate increases in Icatu-WW plants above 25/20°C (**Figure 3B**).

Stress combination did not increase membrane permeability at any temperature in Icatu, and until 37/28°C in CL153, as compared with their respective values of SWD plants at 25/20°C. However, at 42/30°C, significant rises of 49% (compared with SWD at 25/20°C) and 186% (compared with WW at 42/30°C) were observed in CL153-SWD plants. This was in line with the large MDA increases in CL153-SWD plants of 151% (compared with SWD at 25/20°C), and 112% (compared with WW plants at 42/30°C), thus denoting an interaction of stresses at the highest temperature both for leakage and MDA. At 42/30°C, Icatu-SWD plants also showed greater MDA content (77%) than at 25/20°C, but without relevant stress interaction, as no difference was found as compared with the WW plants at 42/30°C.

At Rec14, the SWD plants of both cultivars still showed higher values of membrane permeability and MDA than those of the WW plants at 25/20°C. Yet, only CL153-SWD plants showed greater values than their WW counterparts did at Rec14, pointing to a lower recovery.

### 3.4 Altered activity of antioxidant enzymes

**Figure 4:**
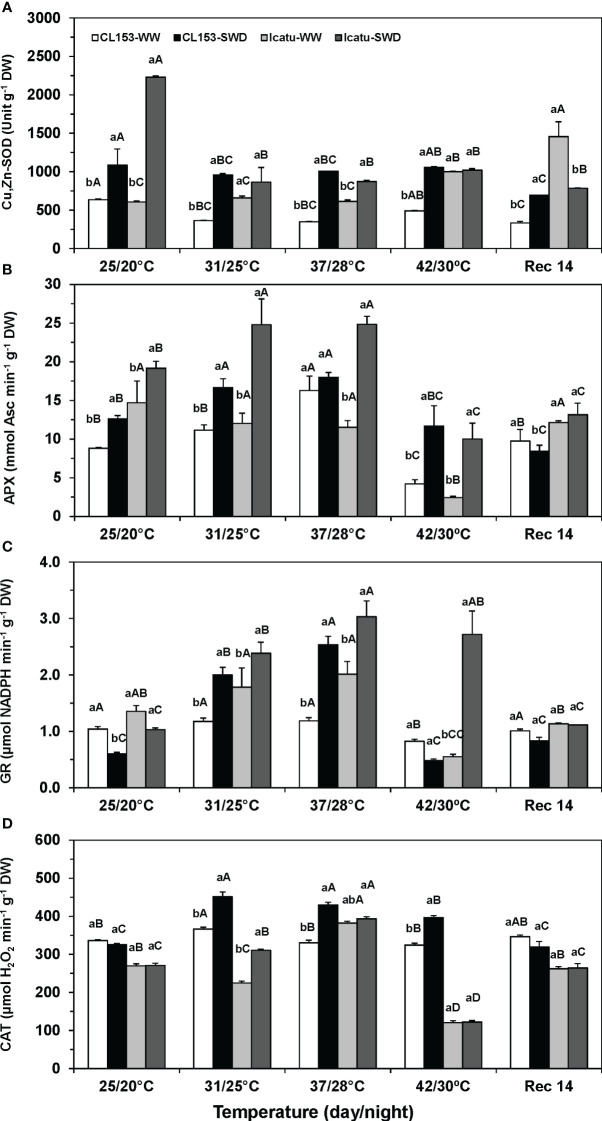
Changes in the leaf maximum apparent activities of **(A)** superoxide dismutase (Cu,Zn-SOD), **(B)** ascorbate peroxidase (APX), and **(C)** glutathione reductase (GR) from chloroplast, and **(D)** cellular catalase (CAT) in *Coffea canephora* cv. Conilon Clone 153 (CL153) and *Coffea arabica* cv. Icatu plants under well-watered conditions (WW) or submitted to severe water deficit (SWD), followed by a gradual temperature increase from (25/20°C, day/night), to 42/30°C, and a recovery period of 14 days (Rec14). For each parameter, different letters after the mean values ± SE (n=4) express significant differences among temperature treatments for the same water level (A–E), or among water availability levels for each temperature treatment (a, b), always separately for each cultivar.

The activities of Cu,Zn-SOD, APX, GR and CAT were influenced by the imposed environmental conditions (**Figure 4**). Under single drought, Cu,Zn-SOD activity increased in both cultivars (71% in CL153; 267% in Icatu) (**Figure 4A**). In turn, temperature raise caused different cultivar impacts, with a declined activity in CL153, including in Rec14, contrasting with significant rises in Icatu under 42/30°C (65%) and Rec14 (172%), always as compared with the 25/20°C value. In CL153 plants, the combined stress exposure barely modified the activities observed for SWD plants at 25/20°C, although showing always greater values than those of WW plants at each temperature. In Icatu, it should be noted a sharp drop in Cu,Zn-SOD activity from 25/20°C to 31/25°C in SWD plants, which was mostly maintained afterwards. Higher values were observed in SWD plants at 31/25°C and 37/28°C than in their WW counterparts, but no difference was observed at 42/30°C. At the end of the recovery period, the activity values approached those of WW plants at 25/20°C in both cultivars, with the already mentioned exception of Icatu-WW plants that displayed a significantly higher value.

Single drought promoted chloroplast APX activity at 25/20°C both in CL153 (43%) and Icatu (30%) (**Figure 4B**). With temperature rise to 37/28°C, the APX activity increased in WW CL153 plants (84%), but did not change in Icatu. However, under 42/30°C, sharp activity declines of 52% (CL153) and 84% (Icatu) were observed, as compared to their respective values at 25/20°C. Notably, when submitted to both stresses, CL153 and Icatu plants showed greater APX activities than those of their WW counterparts at each temperature, with maximal values at 37/28°C, especially in Icatu (116% rise). With a further temperature rise to 42/30°C, APX activity globally declined, although greater values in CL153 (+178%) and Icatu (+317%) were found in SWD plants, as compared to their respective WW plant counterparts. At Rec14 all plants approached their control values (WW at 25/20°C).

In contrast to SOD and APX, the GR activity declined under single SWD in both cultivars (**Figure 4C**), particularly in CL153 (-42%), while the single heat (WW plants) impact was cultivar-dependent. Plants of CL153 maintained GR activity along the experiment (with a decline of 21% under 42/30°C), whereas Icatu plants showed a gradual activity increase until 37/28°C (+49%) followed by a large decline by 42/30°C. When exposed to both stresses a synergistic effect was observed in GR activity in both cultivars, with clear increases until 37/28°C (114% in CL153; 51% in Icatu), as compared with the WW plants at the same temperatures. Yet, only Icatu-SWD plants maintained greater GR activity (394%) at 42/30°C than their WW plants. At Rec14, the GR activity values were close to those of the respective WW plants at 25/20°C.

CAT activity (**Figure 4D**) was irresponsive to single SWD in both cultivars. In CL153, this enzyme activity was also maintained with rising temperatures (and in the recovery period). In contrast, Icatu presented a significant activity rise (37/28°C) followed by a decline (42/30°C). Still, CAT values did not differ from control (25/20°C) at Rec14. The combined heat and SWD conditions significantly promoted CAT activity in CL153 under 31/23°C (23%), 37/28°C (30%) and 42/30°C (22%), and only under 31/23°C (38%) in Icatu, always as compared to the respective WW plants at each temperature. Furthermore, in Icatu, CAT was quite heat sensitive since its activity was strongly depressed under 42/30°C (*ca*. 55%) regardless of water availability, as compared to their values at 25/20°C. By Rec14 no differences were depicted between WW and (previous) SWD plants, and between their values as compared to the controls (25/20°C).

### 3.5 Changes in non-enzymatic protective molecules

#### 3.5.1 Photoprotective pigments

For the sake of the presentation of results from this large data set, our attention will be mostly focused on the single effects of drought (SWD, 25/20°C), temperature (37/28°C or 42/30°C, WW), and the simultaneous occurrence of these two conditions (**Table 2**).

**Table 2:** Changes in the leaf content of carotenoids (xanthophylls and carotenes), and the xanthophylls de-epoxidation state (DEPS), as well as the (α/β) Carotene, and (V+A+Z)/Total Carotenoid ratios.

Genotype	Water	Temperature (day/night)				
		25/20 °C	31/25 °C	37/28 °C	42/30 °C	Rec14
Neoxanthin (mg g^-1^ DW)						
**CL 153**	**WW**	0.243 ± 0.010 aA	0.221 ± 0.011 aA	0.206 ± 0.017 aA	0.219 ± 0.013 aA	0.196 ± 0.028 aA
	**SWD**	0.179 ± 0.015 aAB	0.237 ± 0.024 aA	0.197 ± 0.011 aAB	0.214 ± 0.007 aAB	0.159 ± 0.018 aB
**Icatu**	**WW**	0.214 ± 0.007 bAB	0.242 ± 0.017 aAB	0.248 ± 0.008 aA	0.254 ± 0.009 aA	0.205 ± 0.006 aA
	**SWD**	0.283 ± 0.017 aA	0.241 ± 0.015 aAB	0.287 ± 0.050 aA	0.270 ± 0.021 aA	0.199 ± 0.012 aB
Zeaxanthin (mg g^-1^ DW)						
**CL 153**	**WW**	0.047 ± 0.006 bA	0.053 ± 0.007 bA	0.045 ± 0.005 bA	0.070 ± 0.010 aA	0.098 ± 0.021 aA
	**SWD**	0.235 ± 0.047 aA	0.185 ± 0.034 aAB	0.176 ± 0.030 aAB	0.112 ± 0.013 aBC	0.059 ± 0.014 aC
**Icatu**	**WW**	0.048 ± 0.006 bA	0.039 ± 0.004 bA	0.038 ± 0.009 bA	0.091 ± 0.013 aA	0.059 ± 0.008 bA
	**SWD**	0.403 ± 0.034 aA	0.164 ± 0.045 aB	0.101 ± 0.014 aB	0.117 ± 0.023 aB	0.160 ± 0.010 aB
Violaxanthin (V) + Antheraxanthin (A) + Zeaxanthin (Z) (mg g^-1^ DW)						
**CL 153**	**WW**	0.347 ± 0.011 aA	0.300 ± 0.013 bA	0.278 ± 0.021 aA	0.260 ± 0.020 aA	0.337 ± 0.042 aA
	**SWD**	0.394 ± 0.038 aA	0.391 ± 0.054 aA	0.309 ± 0.029 aAB	0.263 ± 0.018 aBC	0.198 ± 0.019 bC
**Icatu**	**WW**	0.360 ± 0.013 bA	0.397 ± 0.020 aA	0.319 ± 0.014 aA	0.318 ± 0.008 aA	0.319 ± 0.028 aA
	**SWD**	0.552 ± 0.033 aA	0.387 ± 0.043 aB	0.339 ± 0.041 aB	0.329 ± 0.031 aB	0.373 ± 0.013 aB
DEPS						
**CL 153**	**WW**	0.246 ± 0.042 bB	0.250 ± 0.024 bAB	0.209 ± 0.031 bB	0.310 ± 0.040 bAB	0.405 ± 0.045 aA
	**SWD**	0.665 ± 0.068 aA	0.525 ± 0.064 aA	0.628 ± 0.045 aA	0.543 ± 0.015 aA	0.282 ± 0.057 aB
**Icatu**	**WW**	0.181 ± 0.018 bBC	0.124 ± 0.017 bC	0.141 ± 0.036 bBC	0.332 ± 0.046 bA	0.243 ± 0.034 bAB
	**SWD**	0.735 ± 0.029 aA	0.437 ± 0.073 aB	0.401 ± 0.043 aB	0.439 ± 0.038 aB	0.515 ± 0.024 aB
Lutein (mg g^-1^ DW)						
**CL 153**	**WW**	0.785 ± 0.033 aA	0.701 ± 0.030 aA	0.639 ± 0.038 aA	0.813 ± 0.044 aA	0.771 ± 0.106 aA
	**SWD**	0.631 ± 0.045 bA	0.669 ± 0.121 bA	0.662 ± 0.041 aA	0.741 ± 0.052 aA	0.608 ± 0.074 bA
**Icatu**	**WW**	0.713 ± 0.025 bC	0.773 ± 0.044 aBC	0.900 ± 0.031 aB	1.136 ± 0.036 aA	0.845 ± 0.050 aBC
	**SWD**	0.928 ± 0.055 aAB	0.763 ± 0.070 aB	0.790 ± 0.040 aB	1.051 ± 0.098 aA	0.832 ± 0.046 aB
α-Carotene (mg g^-1^ DW)						
**CL 153**	**WW**	0.178 ± 0.012 aA	0.170 ± 0.017 bA	0.195 ± 0.027 aA	0.138 ± 0.027 aA	0.048 ± 0.007 aB
	**SWD**	0.157 ± 0.013 aB	0.220 ± 0.038 aA	0.131 ± 0.014 bB	0.156 ± 0.006 aB	0.055 ± 0.006 aC
**Icatu**	**WW**	0.131 ± 0.015 bBC	0.161 ± 0.013 aB	0.226 ± 0.024 aA	0.161 ± 0.015 aB	0.078 ± 0.004 aC
	**SWD**	0.198 ± 0.019 aAB	0.165 ± 0.015 aBC	0.230 ± 0.019 aA	0.137 ± 0.022 aC	0.064 ± 0.006 aD
β-Carotene (mg g^-1^ DW)						
**CL 153**	**WW**	0.190 ± 0.016 aAB	0.218 ± 0.020 aA	0.191 ± 0.018 aAB	0.222 ± 0.017 aA	0.159 ± 0.012 aB
	**SWD**	0.098 ± 0.004 bB	0.142 ± 0.017 bA	0.091 ± 0.003 bB	0.111 ± 0.010 bAB	0.090 ± 0.006 bB
**Icatu**	**WW**	0.206 ± 0.009 aBC	0.252 ± 0.017 aAB	0.266 ± 0.020 aAB	0.291 ± 0.017 aA	0.191 ± 0.008 aC
	**SWD**	0.144 ± 0.005 bAB	0.134 ± 0.005 bB	0.147 ± 0.005 bAB	0.175 ± 0.013 bA	0.134 ± 0.006 bB
(α+β) Carotene (mg g^-1^ DW)						
**CL 153**	**WW**	0.368 ± 0.024 aA	0.388 ± 0.030 aA	0.386 ± 0.042 aA	0.360 ± 0.041 aA	0.155 ± 0.040 aB
	**SWD**	0.254 ± 0.017 bB	0.362 ± 0.052 aA	0.185 ± 0.031 bBC	0.267 ± 0.013 bAB	0.145 ± 0.011 aC
**Icatu**	**WW**	0.338 ± 0.032 aBC	0.413 ± 0.032 aAB	0.491 ± 0.048 aA	0.452 ± 0.031 aA	0.270 ± 0.007 aC
	**SWD**	0.342 ± 0.021 aA	0.299 ± 0.018 bAB	0.377 ± 0.022 bA	0.228 ± 0.046 bBC	0.199 ± 0.011 bC
(α/β) Carotene (g g^-1^)						
**CL 153**	**WW**	1.148 ± 0.116 bA	0.978 ± 0.129 bA	1.022 ± 0.113 bA	0.584 ± 0.083 bB	0.328 ± 0.071 bB
	**SWD**	1.583 ± 0.086 aA	1.500 ± 0.153 aA	1.435 ± 0.144 aA	1.449 ± 0.120 aA	0.610 ± 0.048 aB
**Icatu**	**WW**	0.627 ± 0.060 bAB	0.662 ± 0.067 bAB	0.865 ± 0.084 bA	0.579 ± 0.069 aB	0.417 ± 0.032 aB
	**SWD**	1.360 ± 0.125 aAB	1.233 ± 0.093 aB	1.556 ± 0.102 aA	0.758 ± 0.123 aC	0.470 ± 0.029 aD
Total Carotenoids (mg g^-1^ DW)						
**CL 153**	**WW**	1.690 ± 0.040 aA	1.611 ± 0.073 aA	1.478 ± 0.115 aA	1.652 ± 0.109 aA	1.511 ± 0.174 aA
	**SWD**	1.544 ± 0.092 aA	1.658 ± 0.253 aA	1.390 ± 0.078 aAB	1.484 ± 0.077 aAB	1.150 ± 0.119 bB
**Icatu**	**WW**	1.625 ± 0.037 bB	1.826 ± 0.091 aAB	1.958 ± 0.061 aAB	2.161 ± 0.053 aA	1.639 ± 0.084 aB
	**SWD**	2.104 ± 0.113 aA	1.690 ± 0.130 aAB	1.916 ± 0.240 aAB	1.961 ± 0.170 aAB	1.554 ± 0.071 aB
(V+A+Z)/Total Carotenoids (g g^-1^)						
**CL 153**	**WW**	0.199 ± 0.005 bAB	0.187 ± 0.004 bAB	0.170 ± 0.004 bB	0.157 ± 0.008 aB	0.223 ± 0.010 aA
	**SWD**	0.275 ± 0.018 aA	0.246 ± 0.024 aAB	0.220 ± 0.011 aBC	0.176 ± 0.004 aD	0.184 ± 0.011 bCD
**Icatu**	**WW**	0.221 ± 0.005 bA	0.218 ± 0.005 aA	0.163 ± 0.006 aB	0.148 ± 0.004 aB	0.193 ± 0.009 bAB
	**SWD**	0.264 ± 0.010 aA	0.224 ± 0.011 aA	0.178 ± 0.006 aB	0.168 ± 0.008 aB	0.238 ± 0.009 aA

Results obtained in Coffea canephora cv. Conilon Clone 153 (CL153) and Coffea arabica cv. Icatu plants submitted to well-watered (WW) and severe water deficit (SWD), followed by a temperature increase from (25/20°C, day/night), to 42/30°C, and a recovery period of 14 days (Rec14). For each parameter, different letters after the mean values ± SE (n=5) express significant differences among temperature treatments for the same water level (A, B, C, D), or among water availability levels for each temperature treatment (a, b), always separately for each cultivar.

Regarding single SWD, Icatu showed a much stronger carotenoid response than CL153. In fact, Icatu-SWD plants displayed significant increases of neoxanthin (32%), the pool of xanthophyll cycle (violaxanthin+antheraxanthin+zeaxanthin, V+A+Z) (*ca*. 4-fold), and zeaxanthin (*ca*. 7-fold), with the resulting DEPS rise. Additionally, significant increases in lutein (32%), α-carotene (50%) and total carotenoids (30%) were observed only in Icatu. Furthermore, Icatu-SWD plants consistently presented greater contents of all carotenoids than CL153-SWD counterparts. In sharp contrast to Icatu, only zeaxanthin (*ca*. 5-fold) and DEPS significantly increased in CL153 plants, although without *de novo* synthesis of the xanthophyll cycle pool. Also, CL153 plants presented declines for lutein (20%), and (α+β) carotenes. A decline of β-carotene, and increases of the (α/β) carotenes and (V+A+Z)/total carotenoid ratios, were commonly found in both cultivars.

The single heat exposure promoted a quite different response pattern and to a smaller extent in both cultivars than did drought, although with a greater responsiveness of Icatu-WW at the two highest temperatures, showing significant increases of lutein (59% at 42/30°C), α-carotene (73% at 37/28°C), β-carotene (42% at 42/30°C), (α+β) carotenes (higher than 33% at both temperatures), and total carotenoids (33% at 42/30°C), always as compared to their WW plants at 25/20°C. Also, DEPS values almost doubled at 42/30°C. All the other pigments and ratios were mostly unresponsive to heat stress. In contrast, the only detected significant modification in CL153 was related to the decline of the (α/β) carotene ratio at the highest temperature.

The combined stress exposure promoted the harshest conditions. Overall, this usually worsens a few plant responses regarding carotenoids. In fact, up to 37/28°C the pigments content in the SWD plants of both cultivars did not significantly differ from those observed under the single drought exposure at 25/20°C, except as regards zeaxanthin, V+A+Z pool, and DEPS values, which showed already clear reductions in Icatu-SWD plants. With a further increase to 42/30°C thermal impacts were mostly maintained in Icatu, but were now also found in CL153-SWD plants. At this maximal temperature, β-carotene (and (α+β) carotenes) was particularly affected, being the only pigment to be reduced under the stress combination as compared to the WW plants of both cultivars at 42/30°C (although maintaining similar values to those observed in SWD plants at 25/20°C). Zeaxanthin and V+A+Z did not differ between WW and SWD plants at 42/30°C, showing that the stress combination did not aggravate the impact on these pigments. However, SWD plants showed lower contents at 42/30°C than at 25/20°C, in parallel with a clear decline of the (V+A+Z)/total carotenoids ratio that was minimal at 42/30°C regardless of watering and cultivar, thus denoting lowered photoprotection ability. Notably, at this harsh temperature the chlorophyll content (Chl (*a*+*b*)) was not significantly affected in either cultivar or water condition as compared to 25/20°C, but Chl (*a*/*b*) ratio was reduced (data not shown).

By Rec14, α-carotene (and (α+β) carotene) pools decreased in the WW plants, whereas the contents for all the other pigments, total carotenoids, DEPS (except CL153), and the (V+A+Z)/total carotenoids ratio, were similar to values of WW plants at the beginning of the experiment (25/20°C), without marked differences between cultivars. This pointed to a reequilibrium in only two weeks after control conditions were reestablished, and a high plasticity of fully developed coffee leaves. Still by Rec14, the SWD plants, although following a close pattern to that shown by their WW counterparts, kept declines of β-carotene (further decreasing (α+β) carotene) in both cultivars, as well as lower contents of V+A+Z and total carotenoids, while maintaining higher DEPS (only in Icatu) as compared with the WW plants under control temperature at the experiment start. Overall, these findings taken together denoted a permanence of greater aftereffects in SWD than in WW plants, particularly in CL153 where a 40% decrease in Chl (a+b) content was found (data not shown).

#### 3.5.2 Ascorbate

**Figure 5:**
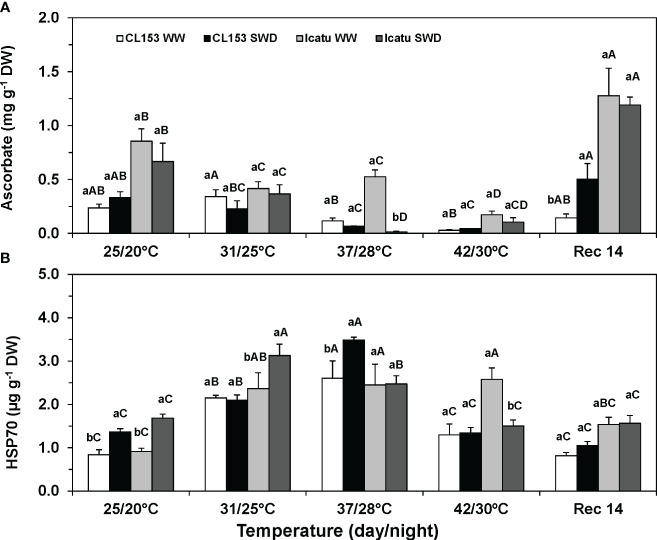
Changes in the leaf content of **(A)** ascorbate, and **(B)** heat shock protein 70 kDa (HSP70) in *Coffea canephora* cv. Conilon Clone 153 (CL153) and *Coffea arabica* cv. Icatu plants under well-watered conditions (WW) or submitted to severe water deficit (SWD), followed by a gradual temperature increase from (25/20°C, day/night), to 42/30°C, and a recovery period of 14 days (Rec14). For each parameter, different letters after the mean values ± SE (n=4) express significant differences among temperature treatments for the same water level (A–D), or among water availability levels for each temperature treatment (a, b), always separately for each cultivar.

Under single drought, ASC contents were not significantly modified in either cultivar, but Icatu presented greater constitutive values than CL153 (**Figure 5A**). Temperature rise promoted ASC declines in both WW and SWD plants at 37/28°C and, especially, at 42/30°C, when minimal values (with decreases above 80%) were observed in both cultivars. Regardless of previous water conditions, at Rec14 the ASC content greatly increased to values close (CL153) or above (Icatu) those at 25/20°C, with rises of 49% (WW) and 39% (SWD) in the latter cultivar.

#### 3.5.3 Heat shock protein 70 kDa

Irrespective of cultivar, HSP70 content significantly increased due to single drought and, especially, single heat (31/25°C and 37/28°C) exposure, with increases of 210% (CL153-WW) and 168% (Icatu-WW) at the latter temperature (**Figure 5B**). However, at 42/30°C, only Icatu-WW plants maintained such increased contents. These stresses superimposition further promoted HSP70 increases to maximal values in SWD plants of CL153 at 37/28°C (*ca*. 154%), and of Icatu at 31/25°C (*ca*. 86%), as compared to the respective values at 25/20°C. Yet, a strong decline was found at 42/30°C in the SWD plants of both cultivars as compared with the values measured at 37/28°C. At Rec14, both cultivars showed HSP70 contents that approached those of WW control plants at the beginning of the experiment.

#### 3.5.4 Main non-structural carbohydrates

Soluble sugars showed interesting alterations as regards single and combined stress exposure, somewhat differently between cultivars (**Table 3**). Drought significantly reduced sucrose content to 17% (CL153) and 24% (Icatu) as compared to the respective WW plants, whereas trehalose was nearly halved in CL153 and fructose was increased in Icatu (by 63%). However, the greatest significant changes were observed in mannitol values that increased 6.2- and 11.6-fold in CL153 and Icatu respectively, with repercussions on total soluble and total carbohydrate contents. Notably, raffinose, glucose and arabinose (both cultivars) and trehalose (Icatu) and fructose (CL153) remained unchanged.

**Table 3:** Changes in the leaf content of non-structural carbohydrates in *Coffea canephora* cv.

Cultivar	Water	Temperature (day/night)				
		25/20°C	31/25°C	37/28°C	42/30°C	Rec14
Raffinose (mg g^-1^ DW)						
**CL 153**	**WW**	0.64 ± 0.26 aB	1.74 ± 0.37 aAB	0.62 ± 0.11 aB	0.78 ± 0.33 aB	2.19 ± 0.56 aA
	**SWD**	0.62 ± 0.23 aA	0.68 ± 0.11 aA	0.11 ± 0.05 aA	0.41 ± 0.13 aA	0.95 ± 0.13 aB
**Icatu**	**WW**	1.1 ± 0.19 aB	0.78 ± 0.3 aB	2.72 ± 0.67 aAB	4.63 ± 1.24 aA	2.03 ± 0.26 aB
	**SWD**	1.06 ± 0.34 aA	1.37 ± 0.54 aA	1.1 ± 0.3 aA	0.28 ± 0.18 bA	1.57 ± 0.1 aA
Trehalose (mg g^-1^ DW)						
**CL 153**	**WW**	3.59 ± 0.94 aA	0.07 ± 0.04 bC	0.94 ± 0.3 aBC	0.33 ± 0.17 aBC	1.84 ± 0.31 aB
	**SWD**	1.49 ± 0.38 bA	1.3 ± 0.17 aA	0.95 ± 0.12 aA	0.64 ± 0.2 aA	0.49 ± 0.12 bA
**Icatu**	**WW**	0.39 ± 0.11 aA	0.49 ± 0.28 aA	0.38 ± 0.24 aA	1.13 ± 0.45 aA	0.7 ± 0.4 aA
	**SWD**	0.58 ± 0.18 aA	0.12 ± 0.08 aA	0.84 ± 0.41 aA	0.7 ± 0.19 aA	0.49 ± 0.14 aA
Sucrose (mg g^-1^ DW)						
**CL 153**	**WW**	20.7 ± 1.2 aA	25.9 ± 1.6 aA	18.1 ± 3.9 aA	19.3 ± 6.3 aA	18.4 ± 1.9 aA
	**SWD**	3.6 ± 0.5 bB	3.9 ± 0.7 bB	2.8 ± 0.4 bB	9.1 ± 2.4 aB	24.1 ± 3.3 aA
**Icatu**	**WW**	25.6 ± 1.8 aB	21.1 ± 0.8 aB	26.7 ± 1.9 aB	17.4 ± 1.8 aB	40.1 ± 3.5 aA
	**SWD**	6.2 ± 0.5 bB	4.1 ± 0.6 bB	7.9 ± 1.5 bB	7.6 ± 0.6 bB	33.8 ± 2.9 aA
Glucose (mg g^-1^ DW)						
**CL 153**	**WW**	0.63 ± 0.11 aA	0.93 ± 0.36 bA	0.42 ± 0.16 aA	0.49 ± 0.1 aA	1.36 ± 0.34 aA
	**SWD**	1.81 ± 0.2 aA	2.32 ± 0.27 aA	1.44 ± 0.37 aAB	0.6 ± 0.1 aBC	0.41 ± 0.07 aC
**Icatu**	**WW**	6.79 ± 1.19 aA	5.82 ± 2.94 aAB	2.1 ± 0.86 bB	1.77 ± 0.17 aB	2.19 ± 0.21 aB
	**SWD**	7.21 ± 0.85 aA	4.16 ± 0.55 aAB	7.36 ± 1.24 aA	2.12 ± 0.57 aB	3.56 ± 0.51 aB
Fructose (mg g^-1^ DW)						
**CL 153**	**WW**	8.43 ± 1.39 aB	8.97 ± 0.58 aB	10.75 ± 0.18 aAB	10.83 ± 0.63 aAB	13.78 ± 0.59 aA
	**SWD**	9.94 ± 0.85 aA	7.84 ± 0.68 aA	8.08 ± 0.38 aA	9.15 ± 0.4 aA	7.87 ± 0.93 bA
**Icatu**	**WW**	5.06 ± 0.25 bA	4.46 ± 0.11 aA	4.46 ± 0.12 aA	4.65 ± 0.21 aA	6.59 ± 0.43 aA
	**SWD**	8.26 ± 0.43 aA	5.37 ± 0.61 aB	5.8 ± 0.49 aB	6.67 ± 0.6 aA	6.62 ± 0.35 aA
Arabinose (mg g^-1^ DW)						
**CL 153**	**WW**	1.41 ± 0.21 aC	2.16 ± 0.2 aBC	3.29 ± 0.3 aABC	3.78 ± 0.51 aAB	4.25 ± 0.44 aA
	**SWD**	2.59 ± 0.42 aA	2.74 ± 0.3 aA	2.28 ± 0.29 aA	1.84 ± 0.3 aA	1.76 ± 0.61 bA
**Icatu**	**WW**	1.38 ± 0.26 aA	0.95 ± 0.24 bA	1.37 ± 0.13 bA	1.26 ± 0.19 bA	1.46 ± 0.11 bA
	**SWD**	2.16 ± 0.33 aC	6.7 ± 0.66 aB	7.87 ± 0.68 aAB	9.7 ± 0.6 aA	8.65 ± 0.66 aA
Mannitol (g g^-1^)						
**CL 153**	**WW**	13 ± 3.6 bB	3.7 ± 1 bB	3.9 ± 0.8 bB	61.1 ± 22.4 aA	24.5 ± 2.8 aB
	**SWD**	94.2 ± 12.8 aA	72.1 ± 8.5 aAB	90.7 ± 7.2 aA	45.3 ± 9.4 aBC	18.7 ± 3.3 aC
**Icatu**	**WW**	8.7 ± 1.2 bA	5.3 ± 0.6 bA	3.2 ± 0.9 bA	5.7 ± 0.6 bA	5.4 ± 0.1 aA
	**SWD**	109.7 ± 6.3 aA	98.4 ± 12.9 aA	65.9 ± 3.8 aB	98.1 ± 2.4 aA	10.3 ± 1.2 aC
Total Soluble (mg g^-1^ DW)						
**CL 153**	**WW**	46.1 ± 5.6 bB	43.4 ± 2.2 bB	38.1 ± 4.7 bB	94.4 ± 18.3 aA	64.5 ± 4.4 aAB
	**SWD**	112.7 ± 12 aA	89.6 ± 8.9 aAB	105.4 ± 6.9 aA	66.3 ± 7.5 aBC	51.1 ± 5.8 aC
**Icatu**	**WW**	48.7 ± 2.6 bA	38.4 ± 2.2 bA	40.6 ± 3.2 bA	35.4 ± 2.4 bA	63.1 ± 4.8 aA
	**SWD**	134.6 ± 5.9 aA	120.1 ± 12.3 aA	96 ± 4 aB	124.5 ± 3.3 aA	64.5 ± 1.8 aC
Starch (mg glucose equivalents g^-1^ DW)						
**CL 153**	**WW**	32.5 ± 1.2 aBC	39.4 ± 2.7 aA	39 ± 2.1 aAB	36.8 ± 2.6 aAB	28.9 ± 1.4 aC
	**SWD**	29 ± 0.3 aAB	28.4 ± 1.1 bAB	32.8 ± 0.8 aA	27.6 ± 0.8 bAB	25.9 ± 0.5 aB
**Icatu**	**WW**	55.2 ± 1.3 aA	37.5 ± 3.7 aBC	44.3 ± 2 aB	41.5 ± 2.6 aBC	35.9 ± 1 aC
	**SWD**	39.4 ± 1.3 bA	36.9 ± 0.5 aA	35.4 ± 0.4 bA	24.6 ± 0.5 bB	34 ± 0.5 aA
Total Soluble/Starch (g g^-1^) *						
**CL 153**	**WW**	1.42	1.1	0.98	2.56	2.23
	**SWD**	3.89	3.15	3.21	2.4	1.97
**Icatu**	**WW**	0.88	1.02	0.92	0.85	1.76
	**SWD**	3.42	3.25	2.71	5.06	1.9
Total carbohydrates (mg g^-1^ DW)						
**CL 153**	**WW**	78.6 ± 4.1 bB	82.8 ± 1.3 aB	77.1 ± 1.6 bB	124 ± 11.8 aA	93.4 ± 5.4 aAB
	**SWD**	141.8 ± 18.1 aA	118 ± 7.2 aAB	140.5 ± 6.9 aA	93.9 ± 10.4 aBC	77.5 ± 7.6 aC
**Icatu**	**WW**	103.9 ± 1.6 bA	75.9 ± 2.6 bC	84.9 ± 0.8 bBC	77 ± 1.6 bC	99 ± 3.2 aAB
	**SWD**	173.2 ± 4.6 aA	159.5 ± 11.9 aAB	129.5 ± 4.4 aBC	148.8 ± 3.6 aB	97.4 ± 2.2 aC

* Calculated using the mean values of total soluble sugars and starch.

Conilon Clone 153 (CL153) and Coffea arabica cv. Icatu plants submitted to well-watered (WW) and severe water deficit (SWD), followed by a temperature increase from (25/20°C, day/night), to 42/30°C, and a recovery period of 14 days (Rec14). For each sugar, different letters after the mean values ± SE (n=6) express significant differences among temperature treatments for the same water level (A, B, C), or among water availability levels for each temperature treatment (a, b), always separately for each cultivar.

The single heat exposure also resulted in modifications of several soluble sugars. Up to 42/30°C in CL153-WW plants only trehalose (declined 59%), arabinose and mannitol (increased 168 and 370%, respectively) were altered. The latter also led to increases in total soluble and total carbohydrate contents. On the other hand, Icatu WW plants presented a clear decline of glucose (74%), and increases in raffinose (3.2-fold) and trehalose (1.9-fold), with an accumulated reduction in total soluble (27%) and total carbohydrate (26%) contents.

Under stress combination, drought (more than heat) was the major response driver (sometimes from 31/25 to 42/30°C). In fact, the values of SWD plants at supra-optimal temperatures were mostly similar to those found under at 25/20°C (single drought exposure) in both cultivars. The exceptions were found in Icatu, in which glucose further declined and arabinose gradually increased from 31/25°C onwards reaching maximal values by 42/30°C (6-fold rise in SWD plants, as compared with WW counterparts at control temperature), whereas a large pool of mannitol was also maintained. In fact, it is noteworthy that at 42/30°C Icatu-SWD plants presented *ca*. 10- and 16-fold greater mannitol pools than their WW plants at 25/20°C or 42/30°C, respectively, and a doubled value than that of CL153-SWD counterparts at maximal temperature.

Upon recovery, some soluble sugars returned to values close to the initial control conditions, as sucrose, glucose (CL153), trehalose, fructose, mannitol (Icatu), and total soluble in both cultivars. Raffinose (both cultivars), fructose and arabinose (CL153), and sucrose (Icatu) maintained greater pools than at the initial control conditions, overall reflecting that the plants were still under recovery by Rec14.

As regards starch, values changed under single drought only in Icatu (a 29% decline), whereas contrasting heat impacts were found between the two cultivars. In fact, in the WW plants, the values increased about 20% in CL153 until 37/28°C but decreased above 25/20°C in Icatu, with declines between 32% (31/25°C) and 20% (37/28°C). Notably, upon the combined heat and drought conditions both cultivars presented a pattern of lower starch values in SWD plants at each temperature, with the greatest absolute differences at 42/30°C. Still, at Rec14 WW and SWD plants showed similar starch contents, and were closer to their initial control values (except for Icatu WW).

The soluble sugars/starch ratio displayed a common genotypic pattern of variation. This ratio was strongly impacted mostly by drought (alone or with heat from 31/25°C onwards) and was nearly kept upon warming (except for CL153 at 42/30°C). At Rec14, this ratio approached the initial conditions although maintaining increased values.

## 4 Discussion

Water restrictions that promote *Ψ_pd_* values below -3.5 MPa are considered to reflect extreme water deficit for coffee trees (Pinheiro et al., 2004; Dubberstein et al., 2020; Semedo et al., 2021), as it was the case here, with *Ψ_pd_* values below -3.7 MPa. In addition, heat constitutes a major response driver for protective plant responses, but causes strong impairments in coffee leaves at both physiological and molecular levels above 37°C (Rodrigues et al., 2016; Dubberstein et al., 2020; Marques et al., 2021; Vinci et al., 2022). Here, not only harsh single drought and heat were implemented, but also their superimposition, a condition that is expected to be even more frequent in natural conditions. For that, SWD plants were exposed to a long-term temperature rise from 25/20°C to 42/30°C, when *Ψ_pd_* reached values close to -4.4 MPa at the two highest temperatures, similarly in both cultivars (see Dubberstein et al., 2020). Therefore, the interplay of mechanisms that prevent damage to the photosynthetic apparatus is essential for plant acclimation in a warmer and dryer environment.

### 4.1 Drought and/or heat impacts at stomata, membranes and PSII photoinhibition levels

The imposed harsh SWD or high temperature (37/28°C and 42/30°C) conditions greatly increased leaf temperature under the single SWD exposure (**Figure 1**), associated with altered stress indexes (**Figure 2**), increases of photoinhibition indexes (**Table 2**), altered membrane selectivity and lipoperoxidation levels (**Figure 3**), thus in line with the previous reported loss of PSII photochemical efficiency (Dubberstein et al., 2020). Also, under 42°C, expressed genes related to the PSII and PSI reaction centers were usually down-regulated in Icatu, contrary to CL153 where they were up-regulated (Marques et al., 2021). Nonetheless, these impacts on the functioning of the photosynthetic apparatus evince marked post-transcriptomic differences between cultivars as regards each stress, with Icatu often showing greater physiological resilience than CL153 (Martins et al., 2016; Rodrigues et al., 2016).

More in detail, proximal thermal sensing technologies, considering the temperature difference between the leaf and its surrounding environment (ΔT), are indicative of evaporative cooling through transpiration (Tr), with ΔT being expected to rise due to stomata closure (that reduces Tr) as stress severity increases. This approach have been widely used to monitor crop photosynthesis and water use of leaf, plant and canopy in several species (Prashar and Jones, 2016, Mulero et al., 2022), and microenvironment suitability for the coffee crop (Craparo et al., 2017). The severity of the imposed conditions was reflected in the colour analysis (**Figure 1**), in the rise of CWSI and in the decline of I_G_ values (**Figure 2**) under drought or/and heat stress, given that the higher the stress degree results in greater CWSI and lower I_G_ (Jones and Grant, 2016). Using these indexes, drought impact was found to be closely associated with strong reductions of g_s_ and *Ψ_pd_* (Costa et al., 2013), and greater ABA content in *Coffea* spp. (Semedo et al., 2021). Furthermore, I_G_ may be advantageous over CWSI, since it decreases nearly linearly with decreasing g_s_ (Jones and Grant, 2016; Prashar and Jones, 2016). In fact, the maintenance of g_s_ at very low values from 25/20°C to 42/30°C in SWD plants of both cultivars (Dubberstein et al., 2020) justifies both the low I_G_ values and an absence of significant variation of I_G_ (and CWSI) indexes between temperatures. Additionally, quite low g_s_ values were observed in the CL153-WW plants under 42/30°C (Dubberstein et al., 2020), thus justifying the I_G_ (minimal) and CWSI (maximal) values at that temperature. In addition, Icatu-WW counterparts showed high g_s_ values under such temperatures (Dubberstein et al., 2020), thus partly agreeing with the moderately lower impact and smaller differences of I_G_ and CWSI to their control plants (WW at 25/20°C). However, the absence of significant differences in these indexes between WW and SWD plants at 42/30°C, alongside the strong impact in net photosynthesis rates (Dubberstein et al., 2020), pointed to a limitation of these thermal indexes when additional impacts, apart from those associated with stomatal closure, are involved. In fact, coffee leaves usually show intrinsic low g_s_ values, turning stomatal limitations the major constraint to photosynthesis more than mesophyll or biochemical ones under control and moderate stress conditions (DaMatta et al., 2019; Martins et al., 2019). However, under progressively severe drought/heat stresses, non-stomatal constraints will gradually become dominant, as confirmed by the negative impacts on the PSII photochemical efficiency (F_v_/F_m_, F_v_’/F_m_’), photochemical use of energy (Y_(II)_, q_L_) and PSII inactivation (F_s_/F_m_’) in SWD plants of both cultivars (Rodrigues et al., 2016; Dubberstein et al., 2020; Semedo et al., 2021). Hence, CL153 showed greater sensitivity of PSII function to single SWD than Icatu, being the only one to show a *PI_Chr_* rise (**Table 2**) and a decline in the photochemical efficiency of PSII (Dubberstein et al., 2020). Photoinhibition of PSII tend to increase when the light energy absorbed by LHCII pigments exceeds the capability for its photochemical use, as would be the case due to a strong stomata closure (Dubberstein et al., 2020). This might implicate ROS overproduction that potentially causes oxidative damage to cellular components (Wang et al., 2018). Therefore, the impacts in PSII functioning (*PI_Chr_* and F_v_/F_m_) suggest only a partial protection of the photosynthetic machinery against ROS (Martins et al., 2016), and denote a degree of PSII photoinhibition that may reduce the photosynthetic electron flux and even total plant growth (Tikkanen and Aro, 2014; Wei et al., 2018). Such greater impact of the photosynthetic machinery in CL153-SWD plants agrees with the decline of several thylakoid electron carriers (Semedo et al., 2021), as well as with the exacerbation of membrane leakage, reflecting altered membrane selectivity, stability and integrity (Elbasyoni et al., 2017), and has been used to identify tolerant coffee plants to environmental constraints (Fortunato et al., 2010; Peloso et al., 2017; Ramalho et al., 2018). Despite leakage rise, the stability of MDA content, a secondary end-product of the oxidation of polyunsaturated fatty acids that is a proxy of oxidative lipid damage (Dias et al., 2010; Toscano et al., 2016), showed that SWD did not promote membrane lipoperoxidation in either cultivar (**Figure 3**).

As mentioned before, the single temperature rise had no relevant impact on the photoinhibition indexes until the severe temperature of 39/30°C, denoting a quite relevant heat resilience of the coffee photosynthetic machinery, specifically of PSII functioning, and pointing to a tolerance to a higher temperature than the diurnal 37°C previously reported (Martins et al., 2016; Rodrigues et al., 2016; Dubberstein et al., 2020). Additionally, at 37°C only minor transcriptomic impacts where observed, in sharp contrast with the clear differentiation in the number of genes with altered expression in these cultivars at 42/30°C (Marques et al., 2021). In fact, a strong impact in WW plants was observed at this temperature in *PI_Chr_* (and *PI_Tot_*) (greater in Icatu), as well as in CWSI and I_G_ (greater in CL153), in parallel with an F_0_ rise together with an F_v_/F_m_ decline in both cultivars (Dubberstein et al., 2020). The latter two parameters have been used to estimate crop tolerance to high temperature as they can reflect the uncoupling of LHCII from the PSII reaction center (Ruban, 2016), showing that a threshold of tolerance against irreversible PSII photoinhibition was surpassed (Pastenes and Horton, 1999; Baker and Rosenqvist, 2004) at 42/30°C, thus in line with previous findings for coffee plants (Martins et al., 2016; Rodrigues et al., 2016; Dubberstein et al., 2020). However, the preservation of membrane stability is a crucial feature to stress tolerance (Elbasyoni et al., 2017), and thylakoid membranes are considered highly sensitive to heat, with impacts on photochemistry being among the first indicators of sensitivity, with damages occurring at PSII and chloroplast ultrastructure (Mano, 2002). In this context, it is notable that MDA and electrolyte leakage did not change up to 37/28°C, and that even at 42/30°C the membrane selectivity was barely affected in the WW plants of both cultivars (**Figure 3**), revealing an important heat tolerance feature. Still, at the highest temperature, lipoperoxidation and *PI_Chr_* increased in Icatu, in line with the reported rise of the quantum yield of non-regulated energy dissipation in PSII (Y_(NO)_) (Dubberstein et al., 2020), thus suggesting a moderate heat sensitivity of Icatu WW plants at the highest imposed temperature, despite the up-regulation of heat shock protein binding genes under 42°C in Icatu plants (Marques et al., 2021).

Great resilience was further observed in both cultivars under the harshest combined stress conditions. In fact, in SWD plants, thermal indexes, permeability and MDA levels (up to 37/28°C), and photoinhibition indexes (up to 39/30°C), showed an absence of stress interaction, without an aggravated status of SWD plants, as compared with 25/20°C. Membrane stability under stressful conditions was previously reported in coffee plants (Martins et al., 2016; Ramalho et al., 2018; Rodrigues et al., 2018), and was closely associated with several mechanisms (namely in Icatu), including the capability to promptly remodel their membrane lipid matrix (*e.g*., alterations in degree of saturation of fatty acids and lipid classes) (Scotti-Campos et al., 2019), the presence of thermal dissipation mechanisms and CEF involving both photosystems (Dubberstein et al., 2020; Semedo et al., 2021), and the strengthening of antioxidative mechanisms (Fortunato et al., 2010; Martins et al., 2016; Ramalho et al., 2018).

With a further increase to maximal temperature, only the CL153-SWD plants reflected a stronger impact under stress combination (in comparison to their WW plants at 42/30°C or with their SWD plants at 25/20°C), reaching the highest leakage and MDA values. These impacts were accompanied (as in Icatu) by declines in PSII functioning, thylakoid electron transport and carriers content, and RuBisCO activity (Dubberstein et al., 2020), consistent with an overproduction of ROS (Logan, 2005; Nishiyama and Murata, 2014; Gururani et al., 2015). Still, CWSI and I_G_ indexes were maintained, *PI_Dyn_* (and *PI_Tot_*) increased. Also, only in Icatu-SWD plants the *PI_Chr_* declined as compared with WW plants at the same temperature, together with a reduction in Y_(NO)_ (Dubberstein et al., 2020), thus reflecting a lower negative uncontrolled energy dissipation at PSII (Huang et al., 2011).

Overall, despite some impacts of the combined drought and heat exposure, these findings point to a relevant resilience of the studied cultivars to SWD and/or heat up to 37/28°C, namely as regards membrane selectivity and lipoperoxidation (even by 42/30°C, especially in Icatu). These are in line with the absence of Y_(NO)_ rise (thus without relevant negative uncontrolled energy dissipation at PSII) (Pinheiro and Chaves, 2011; Dubberstein et al., 2020; Semedo et al., 2021), and the preservation of thylakoid electron transport events at both PSI and PSII, which are membrane-based events directly affected by thylakoid membrane dysfunction, which in turn reduces photosynthesis (Nouri et al., 2015), and point to a somehow unusual and unexpected resilience to such harsh environmental conditions.

Considering that coffee leaves life spam can reach 1.5 years, depending on position on plant canopy, branching order and season of leaf emission (Rakocevic and Matsunaga, 2018), it is notable that these recently mature leaves, from plants exposed to prolongued and severe stressfull conditions for more than 2 month, presented a notable recovery of several parameters by Rec14. That was the case of I_G_, CWSI and photoinhibition indexes (**Figure 2**; **Table 2**), accompanying the recovery of P_n_ and A_max_ (Dubberstein et al., 2020). Additionally, high tolerance seemed to be also associated with irrelevant (or absent) hydraulic conductivity failure and xylem vulnerability to embolism. In fact, under field conditions coffee trees can spend several days for completely recovering from severe drought stress, which has been ascribed to lack of full recovery in water potential and stomatal conductance, facts which have been shown to be accompanied by intense leaf shedding (Martins et al., 2019). As mentioned, the studied plants showed a full recovery of predawn water potential by Rec4, a gradual increase of g_s_ until Rec14 after resuming irrigation (Dubberstein et al., 2020), and leaf leaf senescence was negligible (if any), and none of the used plants died during the stress period or in the subsequent recovery period. Have saying this, a closer look showed that aftereffects persisted to some extent in leakage and MDA in both cultivars, especially in plants submitted to the stress combination. In fact, photodamage caused by stress superimposition can result in the inability of plants to fully recover PSII function (Zhou et al., 2019). That was the case in our plants that, although maintaining relevant performances/values, denoted a few incomplete recoveries as regards electron transport at both PSs, the contents of thylakoid electron carriers (Cyt *b_559LP_*, Cyt *f* and Cyt *b_563_*), the predictor of the rate constant of PSII inactivation (F_s_/F_m_’), as well as F_v_/F_m_ (Dubberstein et al., 2020). Still, the latter might be related to some extent with the greater presence of photo-dissipation zeaxanthin (and DEPS) in Icatu (**Table 1**) as compared with WW plants both at Rec14 and at the beginning of the experiments.

### 4.2 Photoprotective response to drought and/or heat

Drought alone (and partly in combination with heat at 42/30°C) was the major driver for carotenoid changes, as particularly noted in Icatu. These plants responded to SWD with a global *de novo* synthesis (except β-carotene), as reflected in total carotenoid rise and including zeaxanthin (and DEPS), the pool of the xanthophyll cycle and lutein (**Table 1**), thus greatly improving their photoprotective capabilities. In fact, carotenoids, among them zeaxanthin and lutein, have a crucial photoprotective role in all oxygenic photosynthetic organisms, by acting as ^3^Chl^*^ and ^1^Chl^*^ quenchers, and removing epoxy groups from the oxidized double bonds of lipid fatty acids of chloroplast membranes (Smirnoff, 2005; Pérez-Bueno and Horton, 2008; Demmig-Adams et al., 2022). Therefore their increases in response to the stressful conditions indicate a reinforced protective capacity of *LHCs* in both PSs against excess excitation energy, likely resulting in a decreased formation of highly reactive ^3^Chl and ^1^O_2_, also protecting the PS functioning and Cyt *b_6_/f* complex from photodamage caused by ^1^O_2_, as previously suggested under heat and drought conditions (Martins et al., 2016; Rodrigues et al., 2016; Ramalho et al., 2018). Notably, the decline of β-carotene under SWD in both cultivars suggest a lower ability to protect the Cyt *b_6_/f* activity (Zhang et al., 1999), but this thylakoid complex was impacted by SWD only in CL153 (Dubberstein et al., 2020). CL153-SWD plants presented a decline in most carotenoids, but not in zeaxanthin, V+A+Z pool, and (V+A+Z)/total carotenoids ratio. Yet, in SWD both cultivars did not show increased lipoperoxidation (**Figure 3**), maintained low *PI_Chr_* levels (although increasing in CL153), and increased *PI_Dyn_* values (**Table 2**), agreeing with the fact that zeaxanthin can efficiently prevent lipid peroxidation (Demmig-Adams et al., 2022), acting together with the triggering of other protective mechanisms (see below).

Greater heat tolerance was also associated with increased carotenoid levels, namely zeaxanthin and lutein, in coffee leaves (Martins et al., 2016; Vinci et al., 2022). Here, temperature rise had a much lower global impact on carotenoid changes than did single drought in both cultivars, except for β-carotene. Again, CL153 was largely unresponsive as regards several pigments (neoxanthin, zeaxanthin, V+A+Z, lutein, carotenes) at 37/28°C and/or 42/30°C, whereas Icatu presented relevant increases in lutein, β-carotene, and DEPS at these temperatures. This reflects a greater capability to reduce the excess of excitation energy through thermal dissipation, protecting the photosynthetic machinery when the photochemical use of energy is strongly depressed (Dubberstein et al., 2020). Therefore, as for drought, different genotypic heat responses were observed, but with low impact on membrane permeability and lipoperoxidation (**Figure 3**), despite the increase in *PI_Chr_* (**Table 2**) in both cultivars at 42/30°C.

The combined impact of both stresses (SWD, 42/30°C) only marginally raised the contents of zeaxanthin (and DEPS value), as compared to their WW plants at the same temperature. Additionally, losses of β-carotene (compared with the WW plants at 42/30°C), or zeaxanthin (compared with the SWD plants at 25/20°C) might turn such protection less efficient, in line with the impairments of PSII functioning (F_v_/F_m_) at the highest temperature in contrast with the minor to moderate impacts up to 37/30°C (or even 39/30°C) previously reported in both cultivars (Dubberstein et al., 2020). However, the maintenance of increased content of lutein in Icatu, and its rise from 37/28°C to 42/30°C (also for β-carotene) denoted an additional photoprotective response under the pressure of both stresses, surely complemented by their somewhat greater response of the antioxidative system. This likely contributed to the lower level of lipoperoxidation and leakage (**Figure 3**), as well as to the modest decline of the potential activity of PSII and PSII under the combined stress exposure (Dubberstein et al., 2020), as compared with CL153 plants.

The maintenance of total Chl content (data not shown) further supports the claim of an important resilience of the photosynthetic machinery under these harsh conditions in both cultivars. This points to minor disorganization at the antenna complex level, contrasting to the impact often observed upon exposure to heat (Cui et al., 2006; Lamaoui et al., 2018), drought (Ghassemi et al., 2019) and cold (Haldimann, 1999), which can trigger Chl decline in sensitive cultivars. Additionally, heat stress was reported to increase the Chl (*a*/*b*) ratio in sensitive *Festuca arundinacea* (Cui et al., 2006), contrary to our findings. Here, Chl (*a*/*b*) decline (without a reduction of total Chl) pointed not to a selective Chl *a* loss but rather to a preferential synthesis of LHCII (instead of PSII cores) that contains the majority of Chl *b*, thus concurring for a lower Chl (*a*/*b*) ratio (Kitajima and Hogan, 2003). Yet, the Chl (*a*/*b*) ratio increase was observed in heat tolerant cultivars of other crops (Bita and Gerats, 2013). Therefore, since the Chl (*a*/*b*) ratio decline in both cultivars at 42/30°C was aggravated by SWD, accompanied by higher MDA values (**Figure 3**) and a moderate impact on PSs performance (as in F_v_/F_m_, Dubberstein et al., 2020), a certain susceptibility to these harsh conditions should not be discarded in both cultivars.

Notably, by Rec14, the Chl (*a*/*b*) ratio remained at lower levels together with a reduced content of total Chl (CL153) and α-carotene (both cultivars), but with most carotenoid pools close to their initial control values. This was somewhat surprising since we dealt with recently mature leaves which, therefore, denote a great plasticity in terms of stress responses regarding the photosynthetic pigments. Yet, reduced β-carotene (both cultivars) and, especially, greater zeaxanthin values (Icatu) were observed in the plants previously submitted simultaneously to both stresses, denoting the need for greater energy dissipation capability, as the case in cold sensitive cultivars of *Zea mays* that retained greater amounts of zeaxanthin than chilling-resistant ones, while maintaining lower pools of carotenoids and Chl (Haldimann, 1999). Also, under the decline of total Chl (CL153), the reduction of Chl (*a*/*b*) ratio can be interpreted as indicative of a preferential Chl *a* loss in PSI and impacts on the PSI complex, associated with PSII inhibition, as in the case of coffee trees exposed to high irradiance (Nunes et al., 1993) and cold (Batista-Santos et al., 2011). Altogether, despite the relevant resilience of these plants, and a clear recovery of some parameters already in Rec4 (*e.g*., *F_0_* and *F_v_’/F_m_’*) (Dubberstein et al., 2020), our findings suggest that some aftereffects remained at two weeks after stress ending. This was clearer in the plants previously submitted to both stresses, namely due to their greater zeaxanthin (Icatu) and lower total Chl (CL153), thus in line with the aftereffects revealed by the incomplete recoveries of P_n_, Y_(II)_, PSII activity, and Cyt *b_6_/f* complex (associated with PSI functioning) in SWD plants, especially in CL153 (Dubberstein et al., 2020).

### 4.3 Antioxidative enzymes supporting stress plant resilience

Additionally to the carotenoid photoprotection role, the upregulation of detoxifying mechanisms associated with ROS scavenging is crucial for plant stress tolerance (Asada, 2006; Dumanović et al., 2021). With some genotypic differences, Cu,Zn-SOD activity was greatly intensified by drought and heat (**Figure 4**), reflecting a reinforced potential for O_2_^•-^ scavenging, but with H_2_O_2_ formation that is highly toxic and can also be transformed into OH^•^ (Mittler, 2002; Smirnoff, 2005; Halliwell, 2006). Although CAT seemed to be insensitive to drought in our cultivars, as previously reported for Icatu (Ramalho et al., 2018), the strong rise of APX activity is of utmost relevance to detoxify the produced H_2_O_2_. Therefore, both cultivars reinforced the integrated Cu,Zn-SOD and APX action (but not of CAT and GR) in response to drought, although greater in Icatu for both enzymes, in line with their greater abundance of SOD and APX2 proteins than in CL153 (Marques et al., 2022b).

Supra-optimal temperatures can constitute a strong response driver of APX, CAT, and GR (but not of SOD) activities in some *C. arabica* cultivars (Vinci et al., 2022). This highlight a cultivar-dependent response, since our findings revealed that temperatures up to 37/28°C promoted only APX (CL153) and GR (Icatu) activities, which declined at 42/30°C for both cultivars, as also found in Martins et al. (2016). APX decline might have been compensated in CL153 by the absence of impact on CAT activity throughout the experiment.

Under stress combination, the SWD plants of both cultivars showed greater Cu,Zn-SOD and APX activities until 42/30°C than their WW counterparts at each temperature. Also, increased APX activity up to 37/28°C and in CAT were found, as compared with 25/20°C, together with greater APX activity than their WW counterparts at 42/30°C, when CAT activity strongly declined in Icatu. This points to a complementary APX and CAT action in Icatu, as also commonly found in stress-tolerant plants (Logan, 2005; Dumanović et al., 2021), including citrus under the combined exposure do drought and heat (Zandalinas et al., 2017). Still, CAT denoted clear genotypic differences, with an increase in CL153 (as compared with WW plants at 42/30°C) and a strong decline in Icatu irrespective of water availability. This pointed to a thermal sensitivity of CAT above 37°C in Icatu, confirming earlier findings (Martins et al., 2016). In CL153 such greater activities might reflect a larger presence of substrate (H_2_O_2_) to APX and CAT (Cassia et al., 2018) under these harsh conditions (SWD, 42/30°C), with the protection being insufficient given the maximal values of leakage and MDA (**Figure 3**). This was the case in heat-sensitive *Brassica campestris* that showed greater impact on the photosynthetic apparatus and the membrane system, associated with high ROS and MDA accumulation (Zou et al., 2017; Wang et al., 2018).

Drought led to a moderate decline of GR activity in both cultivars, but heat and stress interaction (up to 37/28°C) largely increased its activity (**Figure 4**). However, by 42/30°C only Icatu-SWD displayed a reinforced GR activity, with positive implications on the antioxidant plant defense response since this enzyme integrates the H_2_O_2_ detoxification network (Foyer and Noctor, 2011; Bendou et al., 2022). This was also the case in Icatu and *C. canephora* cv. Apoatã plants, in response to drought and/or cold stress (Ramalho et al., 2018).

Overall, up to 37/28°C a relevant coordinated antioxidative response as regards Cu,Zn-SOD, APX/CAT and GR activity involved in ROS detoxification was found, particularly in Icatu, in accordance with a greater proteomic response and abundance of detoxification processes under SWD (*e.g*., SOD and APX2) in Icatu (Marques et al., 2022a,b), and differences in gene expression patterns between Icatu and CL153 (Fernandes et al., 2021). Under the single exposure to 42/30°C, the activity reductions of all enzymes were relatively lower in CL153, in line with a lesser impact on the antioxidative system than in Icatu (Martins et al., 2016), and that under extreme heat CL153 might present a shift of H_2_O_2_ control from APX to CAT. In contrast, under single drought exposure, Icatu showed the greatest potential control of O_2_^•-^ and H_2_O_2_ through the combined Cu,Zn-SOD and APX action. Under stress combination, both cultivars showed an increased ability to ROS control from 31/25°C onwards due to greater activities of Cu,Zn-SOD, APX, GR (only Icatu) and CAT (only CL153), as compared with their WW plants at each temperature. Despite the fact that only a partial protection seemed to occur in CL153 at 42/30°C, as reflected by the greater impact on membranes (lipoperoxidation and leakage), an antioxidative response to stress interaction was triggered, as reported in tolerant citrus cultivars (Zandalinas et al., 2017), and similarly with the findings regarding the concomitant impact of drought and cold in *Coffea* spp. (Ramalho et al., 2018). Notably, by Rec14, with a few exceptions (*e.g*., Cu,Zn-SOD in Icatu-WW plants), enzyme activities greatly approached their initial WW values, denoting in general a reduced need for antioxidative action.

### 4.4 Additional protective molecules

#### 4.4.1 Ascorbate and HSP70

ASC is a quantitatively dominant and potent antioxidant in plant cells, found in all sub-cellular compartments, mainly in the chloroplast stroma where exists predominantly (90%) in its reduced form (Dumanović et al., 2021). Is capable of directly capturing ^•^OH, O_2_^•-^, and ^1^O_2_, and reducing H_2_O_2_ to water via an APX reaction, contributing to protecting the photosynthetic components (Noctor and Foyer, 1998; Smirnoff, 2005). In both cultivars, ASC was barely affected by drought alone, and markedly decreased under heat stress, regardless of watering (**Figure 5A**). Therefore, it is unlikely that ASC had a crucial role in the response to these stresses, as also previously suggested for coffee plants exposed to temperatures above 31°C (Martins et al., 2016) or severe drought (Ramalho et al., 2018). However, such very low ASC values in SWD plants at 42/30°C may have partly resulted from the APX activity, using ASC as substrate without subsequent adequate regeneration in the ASC-GSH cycle or at the PSI level (with ferredoxin action), although PSI was found to maintain relevant activity under these stress conditions (Dubberstein et al., 2020). In addition, it is worth remembering that ASC is also a cofactor for violaxanthin de-epoxidase to form zeaxanthin (Dumanović et al., 2021). Thus, the minimum ASC contents at 42/30°C likely limited zeaxanthin production, in accordance with the relatively low zeaxanthin pools at that temperature (**Table 1**). At Rec14, ASC contents recovered in both cultivars, but in Icatu to values above those of the initial controls. This suggests a need to maintain a reinforced antioxidative capacity or a “vaccine” kind of response.

An HSP70 rise under SWD in both cultivars agreed with the doubled content found in Icatu under drought (Marques et al., 2022b). However, heat stimulated even greater changes, with maximal accumulation up to 37/28°C in both cultivars and water levels. Still, only WW-Icatu plants maintained increased values at 42/30°C (**Figure 5B**). These findings were also in line with studies that reported increases in gene expression (Vinci et al., 2022) and HSP70 synthesis among the earliest responses in *Coffea* spp. to warming, even at quite moderate temperatures (31°C) (Martins et al., 2016), as well as to moderate and severe drought in *Coffea* spp. with the up-regulation of heat shock proteins (Fernandes et al., 2021; Marques et al., 2022b). This points to the involvement of HSP70 in the *Coffea* spp. response to water and/or heat stresses, with this reinforcement being often associated with an increased thermal and drought tolerance (Wang et al., 2004; Wahid et al., 2007; Park and Seo, 2015; Anaraki et al., 2018; Lamaoui et al., 2018). This is related to the HSP70 role in controlling cellular signaling, by assisting in protein folding and preventing irreversible protein aggregation (Bita and Gerats, 2013; Park and Seo, 2015; Wang et al., 2018), and facilitating the translocation and degradation of unstable proteins (Fragkostefanakis et al., 2015). Furthermore, HSFs can function as molecular sensors to ROSs, such as H_2_O_2_, and control the expression of oxidative stress response genes during oxidative stress (Miller and Mittler, 2006; Volkov et al., 2006).

#### 4.4.2 Sugar dynamics and potential roles

Overall, despite a large reduction of net C-assimilation under drought, heat, and especially under both stresses (SWD at 42/30°C) (Dubberstein et al., 2020), total soluble sugars and total carbohydrate increased, especially under drought, with the exception of Icatu under single heat exposure (**Table 3**). These increases in C-compounds often result from the uncoupling between C-demand and supply due to an earlier impact on growth (a major C-sink) than in photosynthesis (the main C-source) and metabolism (Muller et al., 2011). Additionally, soluble sugars rise is frequently associated with starch breakdown that is greatly stimulated under drought (Quick et al., 1989; Chaves et al., 2003; Lee et al., 2008). This could be the case only in Icatu, where the largest starch pool declined under both drought and/or 42/30°C. This points to a remobilization of reserves to release energy, sugars and C-derived metabolites to help mitigate these stressful conditions (Thalmann and Santelia, 2017), and was confirmed by the large total soluble content under SWD (even at 42/30°C). That was also responsible by the large rise in the total soluble sugar-to-starch ratio. These changes occurred to a much greater extent in Icatu than in CL153 plants, particularly under stress combination, which in turn maintained its starch levels. Greater drought resistance was reported in a *Phaseolus vulgaris* cultivar that degraded more starch in the leaves than another drought-sensitive cultivar (González-Cruz and Pastenes, 2012). Still, an overall total carbohydrate content (thus, including starch) was observed under all stress combinations and both cultivars (except in Icatu under 42/30°C in WW plants), indicating a net *de novo* synthesis, despite the mentioned restrictions on C-assimilation.

The total soluble and total carbohydrate contents rise resulted from the cumulative modifications of individual sugars, with emphasis on the acyclic sugar alcohol mannitol that showed by far the greatest responsiveness to drought and/or heat (**Table 3**). Mannitol, which is the most abundant polyol in plants (Loescher, 1987), increased in both cultivars (although to greater contents in Icatu, except under 42/30°C), as found in *C. arabica* cv. Iapar-59 under severe drought but not for heat stress (37°C) (Carvalho et al., 2014). Since mannitol accumulation under biotic/abiotic stress conditions has been associated with greater stress tolerance (Sickler et al., 2007; Carvalho et al., 2014; Saddhe et al., 2021), this compound would integrate the stress response of *Coffea* spp., likely in a broad range and complex defence mechanisms. In fact, sugar alcohols act as osmoprotectant by protecting hydration around membranes and proteins (stabilizing these structures), as ROS scavenger, as photosynthetic apparatus protector (Shen et al., 1997; Chan et al., 2011; Keunen et al., 2013), and as storage compounds and redox agents (Rejšková et al., 2007).

Sucrose accumulation in response to dehydration is usually associated with higher stress tolerance (Chaves et al., 2003; Lahuta and Górecki, 2011) associated with its osmoprotectant function for membrane and macromolecule stabilisation (Rejšková et al., 2007; Saddhe et al., 2021). However, sucrose breakdown, with the posterior use of glucose into glucose 6-phosphate, could be used for polyols synthesis, including mannitol (Saddhe et al., 2021). In fact, a decline was found under SWD in both cultivars, despite the larger presence of sucrose synthase 2 in Icatu-SWD (Marques et al., 2022b). Thus, sucrose decline under SWD, together with glucose reduction upon heat (in WW and SWD plants) in Icatu, would have partly supported mannitol synthesis. Still, the larger extent of mannitol increase than sucrose and starch decline together pointed to a *de novo* synthesis, particularly in Icatu.

Arabinose is also reported to be involved in stress response, having a moderate increase under single drought (both cultivars), and gradually increasing in CL153 up to 42/30°C. Under stress superimposition Icatu showed a strong increase already by 31/25°C and maximal rises and values at 42/30°C. Arabinose is an effective osmolyte and its strong reinforcement was associated with drought tolerance (Živanović et al., 2020). Additionally, arabinose is an important component of several cell wall polysaccharides and many cell wall-localised O- and N-glycoproteins involved in environmental plant response (Nguema-Ona et al., 2014; Zhao et al., 2019), among them extensin, arabinosylated xylans and arabinogalactan proteins that can “plasticize” cell walls. These compounds integrating arabinose are involved in the maintenance of cell wall extensibility providing the necessary structural properties to allow the plant to cope with periods of desiccation and rehydration (Moore et al., 2013) and salt stress (Zhao et al., 2019), which is crucial to maintain cell wall integrity, which is a key feature for plants to acclimate to unfavourable environmental conditions. The accumulation of arabinose in *C. arabica* leaves was associated with changes in the chemical profile of cell-wall polymers under heat stress (37°C) (Lima et al., 2013). Hence, the observed rise in arabinose likely had a positive role under the imposed conditions in *Coffea* sp., with particular emphasis on Icatu upon the combined stress exposure.

Belonging to the RFOs, the trisaccharide raffinose is also an important stress-responsive sugar. The accumulation and increased expression of genes associated with its biosynthetic pathway have been shown to play a pivotal importance to plant-acquired tolerance to various biotic/abiotic stresses, namely to desiccation, cold, salinity, drought, osmotic and oxidative stress (Li et al., 2020; Yan et al., 2022; Sanyal et al., 2023). Here, raffinose content was unresponsive to drought in both cultivars, but greatly rose in Icatu plants at the two highest temperatures, as also reported in *C. arabica* cv. IAPAR-59 under heat stress (Santos et al., 2011). Most important, raffinose is involved in stabilizing thylakoid membranes, contributing to the maintenance of electron transport (Santarius, 1973), thus in line with the absence of negative impact in the electron transport potential in the Icatu-WW plants even at 42/30°**C** (Dubberstein et al., 2020).

Finally, trehalose, a disaccharide sugar that can act as an osmolyte and stabilize membrane lipids (Saddhe et al., 2021), and fructose, constituted the less responsive soluble sugars under the applied stress conditions, thus having limited role in these plant responses.

### 4.5 Prompt and partially delayed stress recovery

After a 2 weeks recovery, most photosynthetic components resumed (*e.g*., net photosynthesis and RuBisCO activity), but some aftereffects in the performance of the photosynthetic apparatus were still noted (*e.g*., PSs functioning, photochemical use of energy, electron carriers) (Dubberstein et al., 2020). In a few cases the SWD plants exposed to heat recovered better than their WW counterparts (*e.g*., PSs and Ru5PK activities, Cyt b_559LP_ and b_563_ contents in CL153; and RuBisCO and Ru5PK activities in Icatu), suggesting some degree of stress cross-tolerance. Still, some incomplete recovery was clear, especially in the plants exposed to the stress combination (Dubberstein et al., 2020). This helps to understand the maintenance of reinforced thermal dissipation mechanisms, zeaxanthin and DEPS (CL153-WW; Icatu-SWD), Cu,Zn-SOD (Icatu-WW), APX and ASC (Icatu-WW and SWD), as compared with initial control values. Moreover, by Rec14, raffinose (both cultivars) and arabinose (CL153-WW; Icatu-SWD) maintained increased values, denoting a reinforced protection, although mannitol, total (soluble) sugars, and total carbohydrate return to contents close to their initial control conditions. Overall, our findings simultaneously denoted a relevant recovery and resilience (in the face of the previous very harsh stress conditions) but also that the recovery process was still ongoing regarding a wide number of processes.

## 5 Conclusions

Thermal sensing technologies and stress indexes (I_G_ and CWSI) allowed a first glimpse of the impacts of the single and combined impacts of drought and heat. Both I_G_ (decrease) and CWSI (rise) variations were associated with reduced g_s_ values in SWD plants of both cultivars at all temperatures. Yet, the absence of clear differences between WW and SWD plants at 42/30°C, associated with strong non-stomatal impacts on the C-assimilation apparatus, points to the need for further/deeper analysis.

The CL153 plants showed greater PSII sensitivity (greater *PI_Chr_*) to single SWD than Icatu counterparts, in line with a rise in membrane permeability. Heat alone marginally affected MDA content and membrane selectivity in WW plants of both cultivars up to 42/30°C. The preservation of PSII functioning was maintained up to 39/30°C, according to the unchanged photoinhibition indexes in both cultivars, but by 42/30°C *PI_Chr_* increased (greatly in Icatu), showing that a threshold of tolerance was exceeded. Interestingly, SWD plants did not show an aggravated status below 42/30°C, and at this maximal temperature *PI_Dyn_* increased. However, CL153 plants showed a negative stress interaction, with maximal impact on membranes (lipoperoxidation and leakage) by 42/30°C, whereas Icatu presented a strong decline of *PI_Chr_* and an almost leakage stability.

A deeper analysis identified several mechanisms that support the resilience to the imposed conditions. Drought (partly in combination with 42/30°C) was the main driver for carotenoid rise almost exclusively in Icatu (zeaxanthin, lutein, neoxanthin). Additionally, both cultivars showed greater activities of Cu,Zn-SOD and APX (but not of CAT and GR), as well as of HSP70, all greater in Icatu. This improved photoprotective and antioxidative capability justified the lipoperoxidation stability, although with leakage increase. Drought promoted starch (Icatu) and sucrose breakdown, together with a large *de novo* synthesis of sugars in both cultivars, with emphasis on mannitol that had been associated with stress tolerance.

Single heat (37/28°C and/or 42/30°C) usually promoted a lower response than single drought. Only Icatu showed carotenoid rises (lutein and β-carotene), whereas the antioxidative enzymes activities increased by 37/28°C only for APX (CL153), GR and CAT (Icatu), and large declines at 42/30°C for both cultivars, except CAT in CL153, and Cu,Zn-SOD in Icatu. Yet, HSP70 greatly increased in both cultivars up to 37/28°C, and even at 42/30°C in Icatu, the latter showing also a greater raffinose presence, both of which with known pivotal roles in plant acclimation.

Under the combined stress exposure no additional carotenoid pool rises occurred (with loss of β-carotene) at 42/30°C, but Icatu kept enlarged lutein levels. Additional protection in the SWD plants of both cultivars up to 42/30°C was reflected in greater Cu,Zn-SOD and APX activities than in their WW counterparts, as well in GR only in Icatu at maximal temperature. In contrast, HSP70 maintained values above those of WW-plants at 25/20°C, maximal at 31/25°C (Icatu) and 37/28°C (CL153), but strongly declining at 42/30°C, when mannitol (and arabinose) pools maintained high levels in Icatu. Overall, Icatu-SWD displayed a potential increased protection under the combined stresses, in line with the drop of PI_Chr_, and low rise of membrane leakage, and an absence of lipoperoxidation increase at 42/30°C.

Altogether, our findings showed great plasticity/flexibility of recently mature leaves, and a high resilience to single and combined severe drought and heat conditions, with a better stress response in Icatu to drought and both stress combination, and both cultivars to heat. Also, a clear recovery of most parameters was observed just a few days after reestablishing control conditions (between Rec4 and Rec14, *e.g*., thermal stress indexes, photoinhibition indexes, most sugars). However, impacts were still detected two weeks after stress removal, mainly in the plants previously submitted simultaneously to both stresses (*e.g*., membrane lipoperoxidation and leakage in both cultivars, zeaxanthin and ASC in Icatu). Although we cannot exclude a “vaccine” type of response, this seems to point to a still ongoing recovery process at Rec14, and the persistence of aftereffects in an important time scale, relevant in terms of repeated stress exposure under future environmental conditions.

## Data availability statement

The original contributions presented in the study are included in the article/supplementary material. Further inquiries can be directed to the corresponding authors.

## Author contributions

AR: Formal analysis, Investigation, Methodology, Supervision, Visualization, Writing – review & editing. IP: Data curation, Formal analysis, Investigation, Methodology, Visualization, Writing – review & editing. AL: Data curation, Formal analysis, Investigation, Methodology, Supervision, Visualization, Writing – review & editing. DD: Formal analysis, Investigation, Methodology, Visualization, Writing – original draft, Writing – review & editing. FL: Conceptualization, Formal analysis, Funding acquisition, Investigation, Methodology, Supervision, Validation, Visualization, Writing – review & editing. IM: Formal analysis, Investigation, Supervision, Validation, Visualization, Writing – review & editing. JS: Data curation, Formal analysis, Investigation, Methodology, Visualization, Writing – review & editing. MR: Formal analysis, Investigation, Methodology, Visualization, Writing – review & editing. PS-C: Formal analysis, Investigation, Methodology, Visualization, Writing – review & editing. EC: Conceptualization, Formal analysis, Methodology, Writing – review & editing. WR: Visualization, Writing – review & editing, Data curation, Formal analysis, Investigation. MCS-C: Formal analysis, Investigation, Visualization, Writing – review & editing. FR: Data curation, Formal analysis, Visualization, Writing – review & editing. FP: Conceptualization, Formal analysis, Investigation, Supervision, Writing – review & editing. FD: Conceptualization, Formal analysis, Investigation, Methodology, Validation, Visualization, Writing – original draft, Writing – review & editing. AR-B: Conceptualization, Data curation, Formal analysis, Funding acquisition, Investigation, Project administration, Supervision, Writing – review & editing. JR: Conceptualization, Data curation, Formal analysis, Funding acquisition, Investigation, Methodology, Project administration, Resources, Supervision, Validation, Visualization, Writing – original draft, Writing – review & editing.
